# Assessing the Mental Health of Fathers, Other Co-parents, and Partners in the Perinatal Period: Mixed Methods Evidence Synthesis

**DOI:** 10.3389/fpsyt.2020.585479

**Published:** 2021-01-12

**Authors:** Zoe Darwin, Jill Domoney, Jane Iles, Florence Bristow, Jasmine Siew, Vaheshta Sethna

**Affiliations:** ^1^School of Human and Health Sciences, University of Huddersfield, Huddersfield, United Kingdom; ^2^Section of Women's Mental Health, Health Service and Population Research Department, Institute of Psychiatry, Psychology & Neuroscience, King's College London, London, United Kingdom; ^3^Department of Psychology, University of Surrey, Surrey, United Kingdom; ^4^Community Perinatal Mental Health Service for Croydon, South London and Maudsley NHS Foundation Trust, London, United Kingdom; ^5^Department of Experimental Clinical and Health Psychology, Research in Developmental Disorders Lab, Ghent University, Ghent, Belgium; ^6^Department of Forensic and Neurodevelopmental Sciences, Sackler Institute for Translational Neurodevelopment, Institute of Psychiatry, Psychology & Neuroscience, King's College London, London, United Kingdom

**Keywords:** acceptability, diagnostic test accuracy, evidence synthesis, fathers, partners, paternal depression, perinatal mental health, screening

## Abstract

**Introduction:** Five to 10 percentage of fathers experience perinatal depression and 5–15% experience perinatal anxiety, with rates increasing when mothers are also experiencing perinatal mental health disorders. Perinatal mental illness in either parent contributes to adverse child and family outcomes. While there are increasing calls to assess the mental health of both parents, universal services (e.g., maternity) and specialist perinatal mental health services usually focus on the mother (i.e., the gestational parent). The aim of this review was to identify and synthesize evidence on the performance of mental health screening tools and the acceptability of mental health assessment, specifically in relation to fathers, other co-parents and partners in the perinatal period.

**Methods:** A systematic search was conducted using electronic databases (MEDLINE, PsycINFO, Maternity, and Infant Care Database and CINAHL). Articles were eligible if they included expectant or new partners, regardless of the partner's gender or relationship status. Accuracy was determined by comparison of screening tool with diagnostic interview. Acceptability was predominantly assessed through parents' and health professionals' perspectives. Narrative synthesis was applied to all elements of the review, with thematic analysis applied to the acceptability studies.

**Results:** Seven accuracy studies and 20 acceptability studies were included. The review identified that existing evidence focuses on resident fathers and assessing depression in universal settings. All accuracy studies assessed the Edinburgh Postnatal Depression Scale but with highly varied results. Evidence on acceptability in practice is limited to postnatal settings. Amongst both fathers and health professionals, views on assessment are mixed. Identified challenges were categorized at the individual-, practitioner- and service-level. These include: gendered perspectives on mental health; the potential to compromise support offered to mothers; practitioners' knowledge, skills, and confidence; service culture and remit; time pressures; opportunity for contact; and the need for tools, training, supervision and onward referral routes.

**Conclusion:** There is a paucity of published evidence on assessing the mental health of fathers, co-mothers, step-parents and other partners in the perinatal period. Whilst practitioners need to be responsive to mental health needs, further research is needed with stakeholders in a range of practice settings, with attention to ethical and practical considerations, to inform the implementation of evidence-based assessment.

## Introduction

Mental health disorders during pregnancy and the first postnatal year (the perinatal period) are common, affecting approximately one in five mothers (1, 2). Partners—including fathers, co-mothers, and step-parents—may themselves experience perinatal mental health difficulties. Between 5–10% of fathers experience perinatal depression and 5–15% experience perinatal anxiety (3–5) and it is increasingly recognized that fathers may also experience post-traumatic stress symptoms following the birth (5, 6). Paternal deaths are not recorded, however, fathers face an increased risk of suicide in the perinatal period (7). Prevalence of perinatal mental health disorders in step-parents (i.e., new partners), co-mothers, trans and gender-diverse parents is unknown. However, emerging evidence suggests higher depression symptoms in step-fathers and in lesbian co-parents (8, 9) as well as potential challenges concerning fear of childbirth where both partners have childbearing potential (10). In addition, there may be some distinct challenges for LGBT+ parents, linked to heteronormative systems, stigma, marginalization, assisted reproduction, and invisibility/social and legal recognition as parents (11–14).

Where mothers (i.e., gestational parents) are experiencing perinatal mental health disorders, their partners may be particularly vulnerable to mental illness. Depression in parents is known to be correlated (4). Accurate figures from clinical settings are limited but small studies have estimated prevalence rates of between 42 and 50% in partners of mothers receiving inpatient care for moderate or severe mental illness (15, 16). This may partly reflect various challenges, for example shared environmental stressors (e.g., housing, finance), managing their own worries about the mother, coping with changing relationships and managing increased childcare and household tasks, alongside their other commitments (17).

The cost of perinatal mental health disorders in mothers has been estimated at £8.1 billion for each annual cohort of births in the UK, with around three-quarters of this cost relating to the short- and long-term impacts on the babies (18). The costs of partners' perinatal mental health disorders are also likely to be substantial given that mental illness in either parent can contribute to couple conflict and poorer child development outcomes, as well as poorer outcomes for the parent (19–21). Evidence shows that the support mothers receive from their partner can be protective against the development of maternal perinatal mental health disorders and, amongst those with disorders, have a substantial impact on their recovery and well-being (22, 23). Where an unwell mother is struggling to meet her baby's needs, the baby's psychosocial and emotional development may be protected by relationships with other caregivers, including the co-parent (24, 25).

In many high-income countries, women's mental health needs are routinely assessed in universal services based on mental health history, current symptoms of psychological distress (depression and, to a lesser extent, anxiety) and in some places, wider psychosocial risk factors (e.g., housing, finance). Although approaches vary, there is now a growing consensus of the benefits of universal psychosocial assessment of women, provided that this be a part of an integrated care model with onward referral pathways (26). In the UK, the National Institute for Health and Care Excellence guidelines (27) recommends using a two-stage identification strategy, first asking ultra-brief questions [the Whooley questions, Arroll “help” question and two-item Generalized Anxiety Disorder tool, GAD-2 (28–30)] and in the event of a positive response, following up with a longer self-report tool [e.g., the Patient Health Questionnaire (PHQ-9), GAD-7, Edinburgh Postnatal Depression Scale (EPDS) (31–33)]. Onward referrals may include a specialist mental health midwife or health visitor (public health nurse working with children under 5 years), a primary care general practitioner (family doctor), primary care adult mental health services, or specialist perinatal mental health services (for those experiencing or at risk of moderate-severe mental health disorder). Health professionals in universal services in the UK (e.g., maternity and health visiting) seek to identify mental health needs at the first formal antenatal contact and early in the postnatal period by using the two-stage identification strategy, and are also encouraged to consider using the questions at every contact as part of a general discussion about mental health and well-being.

Despite the implications of partners' mental health and well-being for parents, their children, and health and care services, their difficulties largely remain undetected and unmanaged. This reflects that partners have not been prioritized in policy (34) and that universal services and specialist perinatal mental health services (where they exist) usually focus on the perinatal mental health of the woman, i.e., the birthing or gestational parent, while little support is available to partners (17). In several high-income settings where there is existing provision for routine mental health assessment (or “screening”) with mothers, researchers have called for this to be extended all partners and parents [e.g., (6, 35–37)]. Notably, in England, the National Health Service (NHS) has made a policy commitment (38) to evidence-based mental health assessment and onward signposting for partners of women accessing specialist perinatal services; specifically, perinatal mental health services and planned new services that will target mental health difficulties related to the maternity experience (e.g., fear of childbirth, perinatal loss, traumatic birth). No equivalent commitment has been made to universal provision.

To guide evidence-based practice, it is important to understand the accuracy of a tool, i.e., its ability to correctly identify cases and non-cases (those with and without the condition), the likelihood of false positives (avoiding unnecessary referral for further support) and false negatives (missing those in need). In the literature on assessment for fathers, evidence points toward gendered aspects of mental health and whether male-specific measures are needed that are not limited to “traditional” symptoms of distress, but instead incorporate different signs, including behaviors (39–41). For example, men may be more likely to acknowledge fatigue and irritability, to withdraw socially, use avoidant/escapist activities (e.g., sports, overworking, excessive time on internet/TV, gambling, alcohol use, reckless behavior), and to display hostility and anger (42–45).

Successful implementation of an intervention (here, assessment) into practice depends on acceptability to both those delivering and those receiving the intervention (46). Therefore, alongside establishing a measure's accuracy, we need to understand the acceptability of both the measure and of the identification strategy more broadly, from the perspectives of parents and health professionals. There are known barriers to seeking and accepting help, both for new and expectant parents, and for men (47–52). Other relevant considerations include the ability of services to both identify and respond to needs, and any possible impact of these assessments on women's care or the couple relationship.

Existing reviews have examined fathers' support needs and preferences, and their experiences as a partner of a woman who is accessing universal perinatal services and specialist perinatal mental health services (50, 53, 54); these have not however explicitly addressed fathers' own mental health assessment. To date there is a strong evidence base on the validity and acceptability of methods to identify maternal perinatal mental health difficulties (55–58). In contrast, there is an identified lack of research on fathers' “perceptions and receptiveness” to “routine mental enquiry or screening” (pp. 2144–5) (59).

To inform research and practice, we conducted a mixed methods evidence synthesis to identify and synthesize evidence, specifically in relation to fathers, co-mothers, step-parents and other partners in the perinatal period, on the following: (i) the performance (diagnostic test accuracy) of mental health “screening” tools, and (ii) the acceptability of mental health assessment in relation to individual tools and more widely. The evidence synthesis was undertaken as part of a series of reviews to inform the production of a good practice guide for specialist perinatal mental health services (17).

## Methods

The mixed methods evidence synthesis comprised of two sub-reviews, respectively, examining diagnostic test accuracy and acceptability, with the findings integrated using narrative synthesis. Searching, study selection, extraction and reporting were guided by systematic methods, as described below.

### Search Strategy

A systematic search was conducted in 2019 using electronic databases (MEDLINE, PsycINFO, Maternity, and Infant Care Database and CINAHL). The search strategy, which is available on request from the first author, was designed with information specialists and developed for use with a series of reviews. The search used a combination of keywords and subject headings for all the following concepts: partners, perinatal period, mental health or psychosocial or relationship. The search was intentionally broad, to enable identification of relevant literature across all of the review areas; prioritizing its sensitivity (ability to find relevant studies), recognizing that this may result in low precision (i.e., retrieving numerous non-relevant studies) (60). The performance of the search strategy was tested using key papers and refined accordingly to improve sensitivity.

The electronic databases search was complemented by backward and forward citation chaining, i.e., respectively, checking reference lists within included studies, and checking subsequent studies that cited the included studies. In preparation for publication, forward chaining was used to check for any relevant papers published since the initial search. Records were imported into referencing software (Endnote version X9) and duplicates removed.

### Study Selection and Eligibility Criteria

Records were initially screened by a team of reviewers (ZD, JD, JI, FB, JS, VS) based on the title and abstract. Recognizing the potential for challenges with inter-rater reliability, reviewers used three categories: obtain in full, discuss, exclude. A second reviewer (either ZD or JD) then checked these decisions and potentially eligible studies were obtained in full. Using the eligibility criteria outlined below, full-text articles were assessed for inclusion by the lead reviewer (ZD) and checked by a second reviewer (JD).

#### Criteria Applied Across Sub-reviews

Studies were eligible if they included expectant or new fathers, co-mothers, step-parents or other partners of gestational parents, regardless of the partner's relationship status, connectedness to the child, or gender. Eligibility was restricted to primary research but unrestricted by study design. Inclusion was restricted to studies that were written in English and published and peer-reviewed in an academic journal; no date restriction was applied. Quality appraisal was used to assess the strengths and weakness of the included studies rather than to determine eligibility for inclusion in the review.

#### Criteria Specific to Accuracy of Mental Health Screening Tools

Diagnostic test accuracy studies measure the performance of an “index test” by comparing its results with the results of a “reference standard.” In this review, the index test (i.e., the test whose performance was being assessed) could be any mental health screening tool, for any type of mental health disorder. The reference standard was required to be a standardized diagnostic interview based on international criteria and therefore considered a “gold standard.” Studies using other forms of clinical judgment or a cut-off point on another tool as the reference standard were excluded. No restrictions were made regarding the mode of assessment. Studies that did not meet eligibility for inclusion concerning diagnostic test accuracy were also assessed for eligibility for inclusion in the acceptability sub-review.

#### Criteria Specific to Acceptability

Acceptability was assessed in relation to specific measures or examining the concept/proposal of partners' mental health assessment more broadly, provided it was a stated focus of the study (e.g., stated aim, objective, or data collection topic). Studies reporting on fathers' experiences more widely (e.g., their expectations of antenatal care, or the experiences of partners of women with perinatal mental health disorders) were excluded, as were studies regarding acceptability of women's perinatal mental health assessment (including those that considered partners' presence or involvement in maternal mental health assessment).

Consistent with the definition of acceptability proposed elsewhere (46), our primary interest was anticipated (prospective) and experienced (retrospective) cognitive and emotional responses of those (potentially) receiving or delivering assessment. This included parents' and health professionals' perspectives, gathered using qualitative methods (e.g., interviews or focus groups) or quantitative methods (e.g., survey methods). Where studies reported on relevant behavioral aspects (e.g., recruitment, drop-out and uptake of assessment), these were also extracted as potential indicators of acceptability but recognizing that they may also reflect other elements (e.g., the research study, practical considerations) (46). To maximize learning, studies examining feasibility of assessment were also included, even if they did not report stakeholders' views. In addition, eligibility was not restricted by study design, enabling qualitative, quantitative, and mixed methods studies to be eligible.

### Data Extraction and Quality Appraisal

Three reviewers (ZD, JD, VS) were responsible for data extraction and quality appraisal, with all accuracy studies independently assessed by two reviewers and 20% of acceptability studies independently assessed. Any disagreements were resolved through discussion. Data on study methodology and methods, findings (including performance characteristics of measures, relevant qualitative and survey findings, and behavioral indicators of acceptability) were extracted and study limitations recorded. Relevant Critical Appraisal Skills Programmes (CASP) tools (61) and criteria appropriate to surveys (62) were used to assess the quality and identify the strengths and weakness across various domains, including aims, design, sampling, data collection methods, data analysis methods, interpretation, findings and value of the research. The QUADAS-2 (63) was used to assess the diagnostic test accuracy studies, including participant selection, index test, reference standard, flow and timing (e.g., time interval, verification bias).

### Synthesis

Narrative synthesis was used to integrate the findings of both sub-reviews in a single narrative, enabled by its compatibility with different types of review questions and a diverse range of included studies (64). Within the narrative synthesis, different recognized techniques were used (65). For example, studies were tabulated, recording extensive details of the findings, then grouped by different characteristics (e.g., aim, participant group and setting) to look for patterns within and between groups. Thematic analysis was applied, following the approach described elsewhere (66), to generate themes across the acceptability studies; these were then refined through team discussions.

## Results

**Figure 1:**
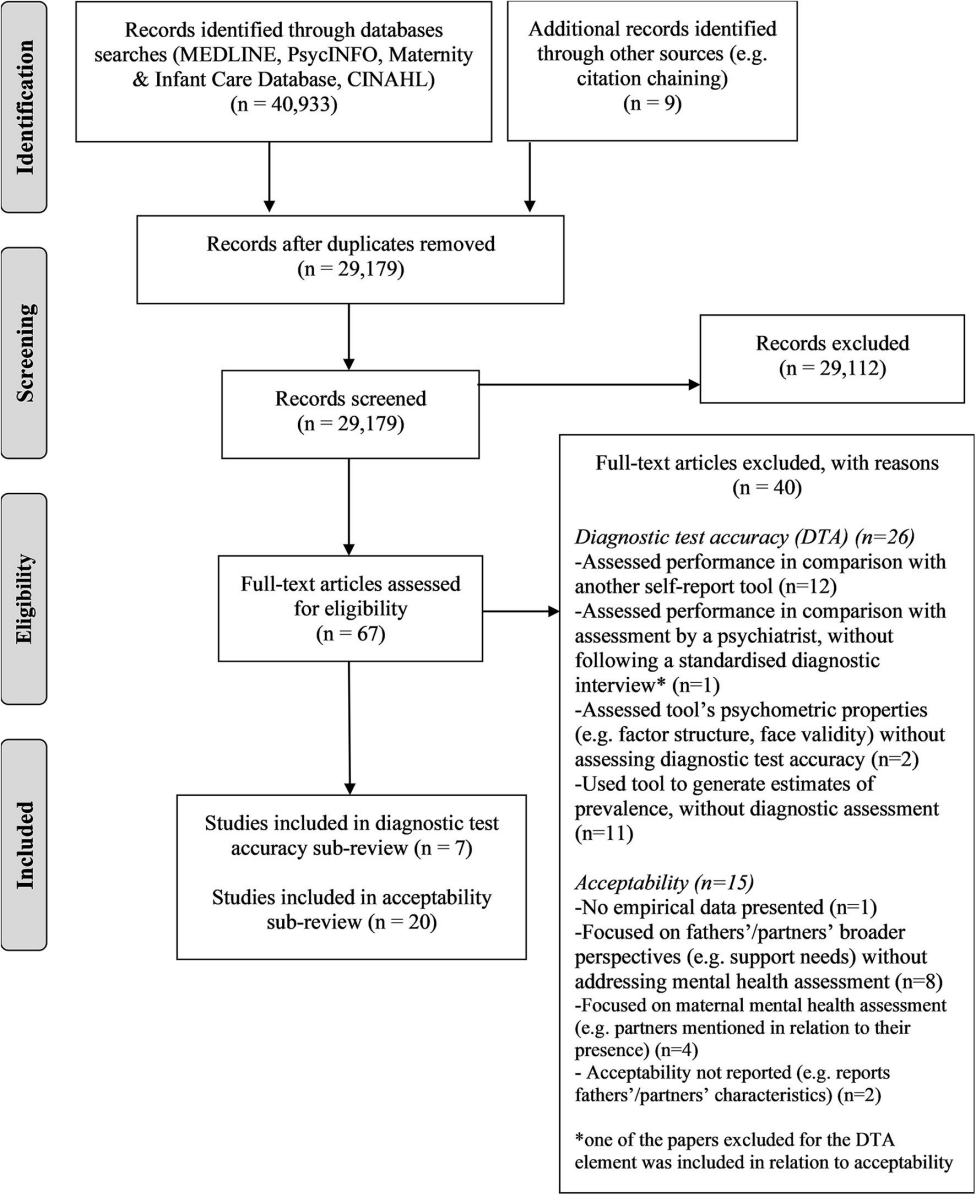
PRISMA flowchart of review process.

The electronic searches identified 40,933 records which were reduced to 29,170 after the duplicates were removed; a further nine relevant references were identified by citation chaining. As shown in Figure 1, screening at the title/abstract level resulted in 67 records being obtained in full, with seven accuracy studies and 20 acceptability studies ultimately being included in the review.

### Overview of Included Diagnostic Test Accuracy Studies

As shown in Table 1, accuracy studies comparing an index test with diagnostic/clinical interview have been conducted in the UK (67, 68), Australia (35), Sweden (69), Portugal (70), Hong Kong with Chinese fathers (71), and Vietnam (72). No studies reported antenatal data separately. Five studies reported postnatal data (6 weeks−6 months) (35, 67–69, 71) and the remaining two used pooled data from antenatal and postnatal timepoints (70, 72), precluding separate estimates.

**Table 1:** Summary of findings of included studies assessing diagnostic test accuracy in fathers (*n* = 7).

**Publication/Country**	**Timing**	**Index test (version)**	**Reference standard**	**Mental health disorder**	**Cases (%)**	**Optimal cut-off**
Areias et al. (70) Portugal	Pooled longitudinal data: antenatal (6 months) and postnatal (3, 12 months)	EPDS (Portuguese)	Schedule for Affective Disorders, regular and lifetime versions	Depression (type unspecified)	12/96 (12.5)	None specified
Ballard et al. (67) UK	Postnatal (6 months)	EPDS (English) (early 13-item version)	Psychiatric Assessment Scale	Depression (type unspecified)	6/48 (12.5)	≥13 EPDS
Edmondson et al. (68) UK	Postnatal (7–14 weeks)	EPDS (English)	Structured Clinical Interview for DSM-IV (SCID), modules for depression and anxiety disorders	Depression (major)	19/189 (10.0)	≥11 EPDS
				Depression (major)/generalized anxiety disorder (GAD)	26/189 (13.8)	≥9 EPDS
Lai et al. (71) Hong Kong	Postnatal (10 weeks)	EPDS, BDI, PHQ-9 (Chinese)	Structured Clinical Interview for DSM-IV, non-patient version (SCID-NP)	Depression (minor/ major)	17/551 (3.1)	≥9 EPDS ≥6 BDI ≥4 PHQ-9
Massoudi et al. (69) Sweden	Postnatal (3–4 months)	EPDS, HADS-A (Swedish)	Primary Care Evaluation of Mental Disorders (Prime-MD), modules for depression and anxiety disorders	Depression (major)	8/262 (3.1)	≥12 EPDS
				Depression (minor/major)	28/262 (10.7)	≥9 EPDS
				Anxiety (type unspecified)	29/262 (11.1)	≥8 EPDS ≥8 HADS-A
Matthey et al. (35) Australia	Postnatal (6–7 weeks)	EPDS (English)	Diagnostic Interview Schedule	Depression (minor/major)	7/200 (3.5)	≥10 EPDS
				“Distress” (minor/major depression, adjustment disorder with anxiety (all criteria for GAD except duration of 6 months), panic disorder, specific phobia)	12/217 (5.5)	≥6 EPDS
Tran et al. (72) Vietnam	Pooled data: spanning antenatal (~28 weeks) and postnatal (~6 weeks)	EPDS, Zung's self-rated anxiety scale (SAS), GHQ-12 (Vietnamese)	Structured Clinical Interview for DSM-IV (SCID), modules for depression, GAD and panic disorder	Perinatal non-psychotic common mental health disorders (including major depression, dysthymia, GAD, panic disorder)	41/231 (17.7)	≥5 EPDS ≥36 Zung SAS ≥1 GHQ-12

*BDI, Beck Depression Inventory; EPDS, Edinburgh Postnatal Depression Scale; GHQ, General Health Questionnaire; HADS-A, anxiety subscale of the Hospital Anxiety and Depression Scale; PHQ, Patient Health Questionnaire; Zung SAS, Zung's Self-rated Anxiety Scale*.

All of the studies recruited through universal settings (e.g., maternity services, health visiting services) and without targeting assessment, for example on the basis of the mother's mental health. In all of the studies, the participants were described as “fathers” or “partners”; all were male and there was only one mention of a non-resident father (68). One study was limited to first-time fathers (35); the others appeared to be mixed regarding parity. Strikingly, the only paper to report ethnicity was the study that was limited to Chinese fathers in Hong Kong (71); this study also contained the widest age range (18–59 years). No studies reported provision of interpreters, with several reporting fluency in the relevant language as an eligibility criterion. Socioeconomic diversity was indicated in three studies (67, 71, 72); elsewhere, high levels of education and employment were indicated, where reported.

Three studies focused on only depression (67, 70, 71); the others additionally examined anxiety disorders, including two that adopted broader approaches of “distress” (including depression, adjustment disorder with anxiety, specific phobia, and panic disorder, although panic disorder was not reported in the paper) (35) and perinatal non-psychotic common mental health disorders (including depression, dysthymia, generalized anxiety disorder, panic disorder) (72). None assessed symptoms of post-traumatic stress.

The Edinburgh Postnatal Depression Scale (EPDS) (31) was assessed in all seven studies (35, 68–73). Other measures assessed come from one Hong Kong study with Chinese fathers (71), validating the Beck Depression Inventory (BDI) (74) and 9-item Patient Health Questionnaire (PHQ-9) (32), one Vietnamese study (72) validating the 12-item General Health Questionnaire (GHQ-12) (75) and Zung's Self-rated Anxiety Scale (76), and one Swedish study assessing the anxiety subscale of the Hospital Anxiety and Depression Scale (HADS-A) (77) (see Table 1). Performance was assessed against the following diagnostic interviews as reference standards: Structured Clinical Interview for DSM-IV (SCID) (68, 71, 72), Schedule for Affective Disorders (70), Psychiatric Assessment Scale (67), Primary Care Evaluation of Mental Disorders (Prime-MD) (69), and Diagnostic Interview Schedule (35).

The characteristics and risk of bias of the diagnostic test accuracy studies are presented in Supplementary Tables 1, 2. Although the majority of studies used consecutive recruitment, it was evident that self-selection bias was a challenge. Time interval between index test and reference test (diagnostic interview) ranged from same-day completion to 8 weeks. Four studies conducted the diagnostic interview with a sub-sample of those completing the index test, sampling by index scores (68, 69, 71, 73). Of these, two provided weighted estimates in recognition of verification bias (68, 69). Most studies reported that assessors of the diagnostic interview were blinded to the results of the index test (35, 69, 71–73); the others were unclear. The only mention of acceptability found in the accuracy studies concerned higher levels of dropout for fathers compared to mothers prior to or during diagnostic interview (35, 70) and a comment that the measures were “acceptable and comprehensible” to participants, with no data reported in relation to this (72).

### Overview of Included Acceptability Studies

As shown in Table 2, all of the 20 studies addressing acceptability were from high-income Westernized countries. Parent perspectives were reported in eight studies (34, 49, 78–83); nine reported health professional perspectives (84–92); none included both. A further three feasibility and implementation studies reported behavioral indicators (e.g., completion rates) without collecting participants' perspectives (93–95).

**Table 2:** Summary of acceptability studies (*n* = 20).

**Publication/Country**	**Aims**	**Design**	**Sample**	**Practice setting**	**Measures**	**Data collection; analysis**
Bagge et al. (82), UK	Acceptability and feasibility of collecting measures with parents of very low birth weight infants in hospital NICU, for research studies	Feasibility study	Fathers (and mothers)	NICU	CES-D, IES-R	Acceptability questionnaire, feasibility data (consent, completion rates), field notes; descriptive statistics
Baldwin et al. (34), UK	Understand men's experiences of first-time fatherhood, their mental health and well-being needs; including support from professionals	Qualitative	Fathers	No	None	Interviews; framework analysis
Clavenna et al. (94), Italy	Feasibility of routine screening with EPDS by family pediatrician at well child visit	Pilot study	Fathers (and mothers)	Pediatric primary care	EPDS	Feasibility data (completion rates); descriptive statistics
Cole et al. (95), USA	Describe implementation of screening parents in specialist hospital NICU by nurses	Implementation study	Fathers (and mothers)	NICU	CES-D, IES-R	Feasibility data (completion rates, processes); descriptive statistics
Currò et al. (93), Italy	Feasibility of routine screening with EPDS by family pediatrician at first well child visit	Feasibility study	Fathers (and mothers)	Pediatric primary care	EPDS	Feasibility data (completion rates); descriptive statistics
Darwin et al. (49), UK	Examine fathers' views and experiences of their perinatal mental health and relevant resources; including mental health assessment	Qualitative	Fathers	No	PHQ-8, GAD-7, PHQ-15 (in original cohort)	Interviews; thematic analysis
Fletcher et al. (79), Australia	Test a set of psychosocial questions with fathers, including ability to identify needs	Mixed methods	Fathers	No	EPDS and 14 questions (e.g., relationships, finance)	Survey and telephone interviews; descriptive statistics
Fletcher et al. (88), Australia	Identify and describe instruments and procedures for screening fathers attending early parenting services, and staff acceptability of screening fathers' mental health	Qualitative	Professionals (clinicians and supervisors/managers)	Early parenting services	Various mentioned	Interviews; thematic survey analysis
Freitas et al. (86), USA (international experts)	Reach expert consensus on the defining factors of paternal peripartum depression; including diagnostics, symptomatology, assessment	Mixed methods	Professionals (practitioners and academics)	No	Various mentioned	Delphi study with online questionnaires; thematic phenomenological analysis and consensus measurement
Greening (78), UK	Assess the “And -how was it for you dad?” questionnaire, designed to encourage men to think about and discuss how they feel, and to promote communication between health visitors and fathers	Pilot study	Fathers	Health visiting	Structured questionnaire including experience of birth and fatherhood	Acceptability questions and feasibility data (completion rates); descriptive statistics
Hammarlund et al. (85), Sweden	Explore child health nurses' experiences of observing depression in fathers during the postnatal period and explore barriers	Qualitative	Professionals (child health nurses)	Child health nursing	None	Interviews; thematic analysis
Massoudi et al. (84), Sweden	Investigate child health nurses' perceptions of working with fathers; including identifying fathers with distress	Survey	Professionals (child health nurses)	Child health nursing	None	Survey; content analysis, descriptive statistics and logistic regression
Oldfield and Carr (89), UK	Explore student health visitors' and newly qualified health visitors' perceptions of their role in supporting fathers when their partner had PND	Qualitative	Professionals (health visitors)	Health visiting	None	Interviews; Interpretive Phenomenological Analysis
Rominov et al. (90), Australia	Describe midwives' perceptions and experiences of engaging fathers in perinatal services	Multi methods	Professionals (midwives)	Maternity	None	Survey and interviews; semantic thematic analysis and descriptive statistics
Rowe et al. (80), Australia	Understand the anticipated needs and preferred sources of mental health information and support of men and women expecting their first baby; including the role of primary care in mental health care	Qualitative	Fathers (and mothers)	No	None	Focus groups (single-sex) and interviews; thematic analysis
Samuel et al. (81), UK	Assess whether prospectively screening parents of children at a PICU for psychological vulnerability to PTSD would enable beneficial targeting of a subsequent follow-up clinic	Randomized controlled trial (all participants were screened)	Fathers (and mothers)	PICU	Post-traumatic Adjustment Screen	Acceptability questionnaire; descriptive statistics
Schuppan et al. (83), Australia	Explore with at-risk men acceptability of screening for paternal mental health concerns and their help-seeking behaviors	Qualitative	Fathers	No	EPDS	Interviews; thematic analysis
Ståhl et al. (92), Sweden	Explore child health services nurses' experiences of performing parental interviews with non-birthing parents	Qualitative	Professionals (child health nurses)	Child health nursing	Whooley questions and EPDS	Focus groups and interviews; content analysis
Wells et al. (91), Sweden	Investigate child health nurses' perceptions of working with fathers, including identifying fathers with distress; making comparisons between 2004 (Massoudi study) and 2014	Survey	Professionals (child health nurses)	Child health nursing	None	Survey; content analysis and various statistics
Whitelock (87), UK	Examine why health visitors do not screen both parents for PND	Qualitative	Professionals (health visitors)	Health visiting	EPDS	Focus groups; thematic analysis

*CES-D, Center for Epidemiologic Studies-Depression scale; EPDS, Edinburgh Postnatal Depression Scale; GAD-7, Generalized Anxiety Disorder Scale; IES- R, Impact of Events Scale—Revised; NICU, neonatal intensive care unit; PHQ, Patient Health Questionnaire; PICU, pediatric intensive care unit; PND, postnatal depression; PTSD, post-traumatic stress disorder*.

Practice-focused studies included studies where assessment was already part of current practice (88) or recently introduced (92), and studies where assessment was introduced into practice in the context of a research study that examined its acceptability and feasibility (78, 81, 93–95). Mental health assessment tools used in practice included the EPDS (88, 92–94), the Whooley questions (28, 92), the Depression Anxiety Stress Scales (DASS) (88, 96), the Center for Epidemiologic Studies-Depression scale (CES-D) (95, 97), the Impact of Event Scale-Revised (IES-R) (95, 98), and the Post-traumatic Adjustment Screen (81, 99). In some services, these were completed as part of a more comprehensive psychosocial assessment (88, 92). In the practice-based studies, acceptability was predominantly examined by completion rates and health professional perspectives (gathered by interview and focus groups) and with little detail reported concerning specific measures. In a further three studies where assessment had not been introduced into practice, fathers were asked to complete specific measures and comment on their acceptability within a research context. This included the EPDS completed away from clinical environments (e.g., home) (79, 83), and the CES-D (82) and IES-R (82), which were completed in a NICU but as a research questionnaire.

All assessments completed in practice settings were postnatal. They included early parenting services that provide support around early parenting difficulties (Australia) (88), services providing special care to infants with health complications or born prematurely (UK and USA) (81, 95), and public health child nursing (Italy, Sweden, UK) (78, 92–94). In contrast, those completed in a research context included completion during pregnancy (79, 83).

The remaining ten acceptability studies reported on views toward partners' perinatal mental health assessment with little or no reference to specific measures and were commonly focused on depression. This included three studies reporting parents' views (34, 49, 80) and seven studies reporting health professionals' views (84–87, 89–91). Some studies had partners' perinatal mental health as their primary focus whereas others reported more widely on engaging fathers in services or on partners' broader support but with specified content that was sufficiently detailed to contribute to the review.

The studies that explored acceptability of partners' mental health being assessed (regardless of measure) commonly used qualitative approaches (interviews and focus groups) (34, 49, 80, 83, 85, 87–90, 92), with a minority using survey methods (84, 90, 91) and one using Delphi consensus techniques with a group of international experts (86).

With the exception of one study that referred to “non-birthing parents” (92), all the studies referred to mental health assessment of “fathers” or “partners.” All partners who participated were male; the majority were resident fathers and in a current relationship with the mother. One study included fathers' and birthing mothers' views (80). Amongst studies involving parent participants, ethnic diversity was indicated occasionally (34, 95), however the majority of studies either did not report ethnicity (78–81, 93, 94) or indicated under-representation of ethnic minority groups (49, 82). Only two studies (93, 95) mentioned the use of interpreters or translation, with most studies limiting participation to parents who were fluent in the relevant language. The majority of health professional participants were female. Professions most commonly represented were health visiting and child health nurses, midwives and psychologists. Further details of the included studies are available in Supplementary Table 4.

### Narrative Synthesis

The synthesis first presents accuracy and acceptability findings related to specific measures, before considering acceptability of partners' perinatal mental health assessment more broadly, grouped across three levels: individual, practitioner, and service.

#### Summary of Findings: Evidence on Diagnostic Test Accuracy of Specific Measures

Several good quality diagnostic test accuracy studies have been conducted with fathers; however, the results are highly varied. The EPDS (31) is the most widely used measure in perinatal mental health research and was used in all the accuracy studies. This review found that it is the only measure to have been validated in the perinatal period in Westernized countries and the only English language version tool to have been validated. Although developed for depression, it has also been used to assess anxiety in mothers (100) and the included studies examined its use for depression, anxiety and categories inclusive of both. However, there is a lack of agreement regarding the cut-point to use in fathers. The highest (≥13) (67) is not comparable due to using the 13-item EPDS which is no longer used. The others recommend: ≥11 for depression and ≥9 for depression/anxiety (68); ≥10 for depression and ≥6 to avoid missing ‘any distress' (including depression and anxiety) (35); ≥12 for major depression and ≥9 for minor/major depression (69); ≥10 for depression (71); and ≥5 for perinatal non-psychotic common mental disorders (72). One study did not specify an optimum cut-point, reporting that the tool was less satisfactory when used with fathers due to poor sensitivity (i.e., under-identification) (70). Where studies assessed multiple tools (67, 69, 71, 72), all concluded that the EPDS performed similarly to, or better than, the other measures assessed (for more details, see Supplementary Table 3).

Some studies considered differences in thresholds across groups within and between studies. The Australian and Vietnamese studies compared thresholds for fathers and mothers and reported lower thresholds were optimal for fathers (35, 72). In contrast, the Swedish study (69) found comparable thresholds for major depression and proposed that their relatively high threshold for fathers, compared with other studies, may reflect there being “no major difference” in how men and women in Sweden express major depression whereas differences may be greater for minor depression, and seen as “more legitimate” for mothers. In finding lower thresholds for fathers than observed in high-income countries, authors of the Vietnamese study (72) proposed that this may reflect firstly cultural differences concerning emotional expression and secondly, that framing questions as symptoms different to their usual state may be insensitive to sustained adversity and poverty found in poorer countries.

Across the included studies, items endorsed varied between fathers who were depressed and those who were non-depressed and across samples. For example, in one study self-harm was endorsed by 50% of the fathers with depression, compared to 5% of non-depressed fathers (71). In contrast, elsewhere self-harm was endorsed by only 3% of fathers and of mothers (69). One study reported that whilst mothers reported significantly more symptoms, the symptoms themselves were similar (67) yet another reported gendered differences in item endorsement, finding no differences for self-blame, sleep difficulties, and thoughts of self-harm but that endorsement of crying was significantly lower in fathers, being reported by only 2.3% (35).

The authors' recommendations concerning the EPDS were divergent. Some recommended its use to screen for depression (71), or positioned that screening fathers for depression may be “valuable” (68); others recommended its use to routinely screen for distress more broadly, i.e., including both depressive and anxiety disorder (35) or to routinely screen for non-psychotic common mental disorders (72). In contrast, one study advised against routine assessment, due to the high number of false positives; instead encouraging targeted use, for example selectively assessing fathers that show signs of distress or when the mother is depressed (69). Furthermore, one study found the tool is not valid for use with fathers due to poor sensitivity, i.e., under-identification (70).

#### Summary of Findings: Evidence of Acceptability of Specific Measures

Although several studies named specific measures, it was rare to report on the acceptability of a measure in detail. Only one study (83) assessed a measure's acceptability in depth, reporting the views of nine expectant fathers with a current or past diagnosis of depression or anxiety, completing the EPDS in a research context. Most reported positive aspects of the measure, finding it relevant and easy to complete, with the phrasing “inviting, comfortable and unintrusive”; however, they also welcomed its anonymity, which would not apply in a practice setting. One study (79) asked expectant fathers to complete the EPDS and some “psychosocial questions” (e.g., relationships) by anonymous postal survey. Acceptability telephone interviews with a subsample (24% of the 29.4% that completed the survey) found that none were “bothered” by any of the questions and they were described “uniformly in positive terms”; however, details were not reported regarding individual questions.

Five studies concerning fathers accessing universal postnatal services (i.e., health visiting or “well child” visits) named specific measures (78, 87, 92–94). One UK study (78) used a questionnaire about fatherhood and the birth experience to encourage discussion of “feelings and emotions” without using specific mental health questions. Asking 20 fathers on the author's own health visiting caseload, all completed the questionnaire; 65% reported the questionnaire was helpful; 60% reported it improved communication with their partner; 20% reported it improved communication with their health visitor (the author); and 85% thought it should be used in future. Comments were not reported regarding individual questions. Another UK study (87) examined health visitors' views on screening fathers for postnatal depression. Most comments concerned assessment more broadly but when asked what prevented them from using the EPDS with fathers, one of the 12 participants reported she would feel comfortable doing this but suggested the need to change some of the words to be more “man-friendly” (87).

A Swedish study (92) reported nurses' perspectives on parental interviews with non-birthing parents where the interview included the use of the Whooley questions (28) and EPDS. Nurses' comments concerned the interview as a whole, rather than the specific measures, with the only relevant comment being that nurses were positive about having a “planned conversational guide,” which they contrasted with previous “loosely organized conversations” even if they had used the EPDS.

Two Italian studies (93, 94) examined feasibility of assessment at universal well child visits with pediatricians, asking fathers to complete the EPDS. One introduced the study at the first visit, seeking consent to complete at the second visit, and found that 38% of fathers completed the EPDS, compared with 73% of mothers (94). The other study reported 99.6% of the fathers (and the mothers) completed the EPDS when conducted as standard practice at the first visit, finding that the EPDS took 2–7 min to complete and that it is feasible to screen fathers with the EPDS in this setting (93).

Within specialist services, four studies named specific measures (81, 82, 88, 95). An Australian study (88) found that early parenting services used a range of tools to screen fathers (including depression, anxiety, psychosocial risk, parenting confidence); the most common being the EPDS but that one service used an adapted version for fathers (details not specified) and another service considered that the EPDS did not effectively screen fathers or mothers for anxiety. The other specialist services concerned neonatal and pediatric intensive care units. One study (82) examined acceptability and feasibility of collecting psychological measures with fathers and mothers in a neonatal intensive care unit (NICU), completed in the context of research. They found ~60% of parents (gender not reported) consented of which approximately half completed and returned the questionnaires; these included measures of trauma (the IES-R) (98) and depression symptoms (CES-D) (97). Acceptability data was not reported by measure but the importance of length was noted, due to time and also the cognitive and affective load for participants. Similarly, another study (95) found that fathers of newborns in specialist-NICU were receptive to screening during the mother's hospitalization (using the IES-R and CES-D), with 79.6% “compliance” (and 96.5% in mothers). In a pediatric intensive care unit setting, screening parents for vulnerability to PTSD [using the Post-traumatic Adjustment Screen (99)] was reported as acceptable to parents, with; 85% of those that went on to complete the questions not reporting any distress in completing the measure; however, only 52% of families consented to complete the questions (81).

#### Summary of Findings: Evidence of Acceptability of Assessment More Broadly

Some fathers voiced that they would like to be asked, or felt they should be asked, about their mental health; others viewed it to be unnecessary or expressed resistance (34, 49, 80). Amongst those who welcomed assessment, some reported feeling excluded by existing provision, and that assessment may help to normalize their experiences and encourage support-seeking; however, this was nonetheless accompanied by ambivalence (34, 49, 80, 83). Health professionals in different settings (including midwifery, health visiting and public health nursing, and early parenting services) viewed fathers' and other co-parents' mental health as important (87–90, 92). Both participant groups (i.e., parents and health professionals) identified factors that influenced their views toward acceptability of assessment and the potential challenges involved. These factors were grouped as candidate themes. Through team discussion and informed by the authors' knowledge of the research literature, including existing reviews on barriers and facilitators to seeking and accepting support in relation to maternal mental health (101–104), it was decided to categorize the themes at the individual-level (including factors influencing families), practitioner-level and the service-level. It is recognized that some may span across multiple levels. The themes are shown in Table 3 and illustrated below, with italics used to denote titles.

**Table 3:** Summary of themes: challenges associated with mental health assessment of fathers, other co-parents and partners.

**Level**	**Themes**
Individual	Gendered perspectives Compromising support for women (birthing parents) Perceived purpose of assessment Ability to recognize symptoms
Practitioner	Knowledge, skills, confidence Fear of causing offense or distress Conflicting needs of parents
Service	Culture of the service Remit of the service Workload and time pressures Opportunity for contact (including lack of privacy, building rapport) Need for training Need for clinical supervision Need for guidelines Need for appropriate tools Need for onward referral routes

##### Individual-Level Influences

*Gendered perspectives* on mental health and help-seeking were indicated in several papers; including in relation to stigma (34, 83) and needing to be “the strong…person,” with mental health difficulties seen as a sign of weakness or vulnerability, threatening masculinity (83). It was suggested that stigma may be overcome by framing information about screening in a way that appealed to men's roles as fathers (83). Health professionals in one study perceived that such barriers may vary across cultures and individual beliefs (87) and another study (88) noted the absence of any comments from professionals about screening of fathers from culturally and linguistically diverse communities. Moreover, in one of the few ethnically diverse samples, it was found that some fathers felt it was culturally and socially unacceptable to discuss difficulties with fatherhood (34). Some fathers were open to discussing their mental health with their partner (79), others noted concerns about completing a tool in their partner's presence (83), reporting concerns about others (e.g., friends, family, colleagues) learning of fathers' mental health needs (34, 83). Some fathers anticipated that the introduction of routine screening would reduce stigma by helping to normalize paternal mental health difficulties (80, 83).

Fathers expressed concerns that women's needs were greater and should be prioritized, and furthermore that partners' assessment may *compromise support offered to women* (34, 49, 83), potentially being a burden to services and being unnecessary (34, 92) and particularly in the context of perceived under-resourced services (49). In addition, some fathers believed that, if assessment indicated that they were struggling, this could be detrimental to the partner, for whom they needed to be seen to be strong (83). The *perceived purpose of assessment* was also relevant; with some fathers indicating their willingness to be screened would depend on the perceived value of completion (80) and that a lack of explanation about “the intention behind and possible outcomes of screening” could increase “stigma, suspicion, and dishonesty” (83), with some fathers perceiving an emphasis on child protection within health visiting services and potential for unwanted involvement (34).

Another factor influencing acceptability was the poor awareness amongst parents of partners' mental health difficulties and *ability to recognize symptoms*. Fathers welcomed more information on signs and triggers (34) and it was noted that greater awareness may reduce barriers to assessment and equally that assessment may raise individual awareness of their own symptoms, prompting help-seeking (83).

##### Practitioner-Level Influences

Professionals' *knowledge, skills, and confidence* was identified both by health professionals and parents as influencing the acceptability of assessment. Some fathers questioned whether primary care providers (across maternity, child and family nursing, and general practitioners) were qualified to support mental health, with their training focusing on physical health (80). Similarly, child health nurses reported lack of awareness of paternal distress and mental health difficulties (84, 91) and health visitors identified a lack of training in theory and in practice on paternal mental health, so felt unable to adequately support fathers. An expert panel reached strong consensus that a psychosocial assessment with fathers should be by someone who understands paternal perinatal mental health (86), indicating that this may currently be perceived as outside the knowledge of many practitioners. Health visitors and child health nurses also shared concerns about not having the skills to support fathers and partners (87, 91). Midwives and health visitors reported lacking confidence, both in working with fathers more generally (89) and in asking them about their mental health (90). In one study, this lack of confidence extended to health visitors expressing fear for their own safety, feeling vulnerable when working alone with men and particularly in the context of mental health difficulties (87).

*Fear of causing offense or distress* was raised by health professionals, who noted the potential for this to be shaped by fathers' individual culture, religion or personal beliefs (87) and some fathers themselves raised that depression screening (here, the EPDS) could challenge those that may want to “avoid difficult feelings” (83). However, where parental interviews with embedded depression screening had been introduced with non-birthing parents in practice, child health nurses described having had only positive responses amongst the parents (here, fathers) who had been offered and accepted the individual interview, but that fathers also expressed surprise at being “included and noticed” (92). Additionally, in a pediatric intensive care setting where parents were asked to complete a trauma measure, of those that subsequently completed the acceptability question, the majority (85%) did not report any distress in completing a trauma measure (81).

In both universal (health visiting and child health nursing) and specialist settings (early parenting services), professionals identified the challenge of *conflicting needs of parents* when working closely with both parents. This included potential “conflict of interest” (89), keeping viewpoints separate, feeling like a mediator and experiencing challenges about managing difficult information and confidentiality; dynamics that would usually be avoided when working with one parent only (92). Tensions could exist when asking mothers about fathers in their absence, including issues of confidentiality or questioning the mother's description (88).

##### Service-Level Influences

There were numerous organization-level influences; many of which were linked to the underlying *culture* and *remit* of the service and in turn shaped attitudes of parents and professionals. The *culture* of services and emphasis on (birthing) mothers was evidenced in universal- (health visiting and child health) and specialist services (early parenting). This included focusing “routines” on mothers (85); making assumptions that the mother would attend child nursing appointments and that the emphasis of communication would be with the mother (84, 91). Linked to this, some child health nurses expressed not seeing men as equal caregivers (84, 91). Furthermore, some health visitors voiced that although viewing as a “mother and child” service could lead to feelings of exclusion amongst fathers, this focus should continue because fewer fathers engage. The prevalence of female staff was also identified as a potential barrier to routinely screening fathers (88).

Linked to the *culture* was the (perceived) *remit of the service*, with some professionals and parents questioning the inclusion of partners' mental health across a range of services (87, 88). Some fathers reported that they would only disclose mental health difficulties if they viewed the health visiting appointment to also be about them (34). A preference was expressed to speak with a general practitioner rather than someone in maternity or health visiting (34, 49); perceiving maternity to be focused on the woman and pregnancy, and physical rather than emotional health (49), with men's emotional well-being not a priority in current models of care (80). Notably, where parental interviews had been introduced with non-birthing parents in child health services, nurses felt their inclusion indicated to society and parents the importance of non-birthing parents, helping them to feel included (92). Within specialist early parenting services, staff reported that a focus on maternal mental health could be a barrier to screening fathers and that the service's function and the father's involvement (e.g., primary caregivers, admitted to the service, actively participating) determined who was screened (88). Rather than being offered routinely, partner assessment may happen in other circumstances, for example, being relevant in the mother's admission, due to health professional concerns, or where the father's mental health was viewed to be relevant to the father-child relationship (88).

*Workload and time pressures* were reported in universal services. Health visitors viewed screening fathers as potentially beneficial but “rejected the proposal” due to caseload concerns (87). Where parental interviews with non-birthing parents had been introduced, some child nurses had been unable to conduct any due to workload and it was recognized that time was essential for discussions to be “possible and meaningful” (92). Fathers similarly perceived health professionals in maternity and health visiting as not having enough time to meet their mental health needs (34, 49).

Commonly reported as a challenge, both by professionals and parents, was the *opportunity for contact* with fathers and other partners (34, 49, 80, 82, 84, 86–89, 91). Services' limited hours and need for flexibility with appointments were raised repeatedly, to accommodate fathers' work commitments and travel time (34, 84, 86, 88). Contact was also seen as related to engagement with services, with fathers participating less in child health care (84); for example, being present at a home visit but choosing to not stay in the room (89). Additionally, child health nurses reported struggling to establish continuity with fathers due to not seeing them regularly (85). Some fathers identified a lack of privacy as a barrier to assessment, feeling unable to talk to a health visitor independently, away from their partner (34); again, linked to the remit and focus of services.

Connected to the *culture* and *remit* of services (service-level), and to the gaps in *knowledge, skills and confidence* (practitioner-level), professionals in universal services (maternity, health visiting and child health) identified a fundamental *need for training* in theory and practice for working with fathers (87) and specifically in relation to paternal mental health (34, 87, 89, 90) and addressing potentially difficult situations when working with couples (92). Professionals with experience of supporting fathers in relation to their mental health identified the importance of access to *clinical supervision* (84, 92).

Across settings, professionals identified the *need for guidelines* as a barrier to assessing fathers' mental health. With no process or guidance in place, some health visitors viewed screening men as problematic (87, 89). Child health nurses reported a range of approaches and lack of structured methods (84), commending the introduction of a planned approach (92). Within specialist services, there was similarly no uniform approach (88) and both professionals and parents expressed that routine screening would help to “normalize” paternal perinatal mental health difficulties (80, 83, 88). Related was the *need for appropriate tools* for use with men (85–88), the *need for onward referral routes* (i.e., mechanisms for referring fathers and other non-birthing parents to appropriate support) (92, 95) and staff having confidence to make these referrals (90).

## Discussion

In the context of growing calls to introduce mental health assessment for partners in the perinatal period (6, 35–37), this mixed methods evidence synthesis sought to address existing research gaps and inform future research, policy and practice. There is clearly significant international interest in using screening tools to identify the mental health needs of partners. Several good quality accuracy studies exist, alongside a range of studies giving some indication of factors influencing acceptability of assessment amongst both partners and professionals. However, it is evident from the included studies that the existing literature is limited in several ways. The vast majority of research concerns resident fathers; no studies examined the perspectives of co-mothers, step-parents or other partners. Although partners can experience a range of perinatal mental health difficulties, the literature is dominated by postnatal depression. Most settings have been universal health visiting or child health services; some have examined acceptability in specialist services where the child has health complications or there are parenting difficulties but no studies have yet examined acceptability in practice in maternity services or specialist mental health services. The acceptability literature is entirely from high-income Westernized countries where maternal mental health assessment is already part of current practice and within these studies, intersections with other factors have been neglected, for example culture, ethnicity, language, education, income.

The EPDS is the measure most assessed, both for accuracy and acceptability. Where studies assessed multiple tools, they all concluded that the EPDS performed similarly to, or better than, the other measures assessed (67, 69, 71, 72). Despite the quality of these diagnostic test accuracy studies, the results are highly varied. Recommendations therefore differ considerably across studies; encouraging routine assessment (35, 71, 72), encouraging targeted use (69), and rejecting the tool's use (70). Where use is recommended, some have argued its use for depression (71) and others for broader categories, of depression/anxiety or distress (35) or non-psychotic common mental disorders (72). In addition, most studies involving fathers were conducted within the first 3 months following birth (35, 68, 69, 71, 72), with few conducted between 6 and 12 months (70, 73). Yet, there is evidence that fathers' vulnerability may peak later than mothers'; for example, depression may be more likely to develop at a later stage for fathers (3–6 months postnatal) (3). Furthermore, the only antenatal data was presented within pooled perinatal data (70, 72); accuracy during pregnancy remains unknown and research with women indicates different thresholds during pregnancy to the postnatal period (105, 106). No studies have yet validated measures of other common mental health difficulties in fathers during the perinatal period against diagnostic interviews. Where trauma has been considered, this has been with parents of children admitted to intensive care, opposed to post-traumatic stress symptoms following childbirth, which are becoming increasingly recognized though not routinely assessed in either birthing or non-birthing parents.

The review identified that ethnicity has been neglected in the existing evidence base on accuracy and differences found between countries indicate the need for further research on cultural influences, both between and within countries. Of note, the studies assessing measures in non-Westernized countries (71, 72) used diagnostic interviews that were culturally appropriate and more able to accommodate alternative expressions. Some studies reported gendered differences in optimal thresholds and in item endorsement between fathers and mothers. Although male-specific measures have been developed to assess depression and mood difficulties (39, 41) and compared with EPDS in the postnatal period (40, 41, 107), none have yet been validated against diagnostic interview. Concerns have been raised however that diagnostic interviews themselves may be subject to inherent gender bias that leads to under-identification in men (108). It must also be acknowledged that many individuals will not identify with these gendered approaches and “gender-inclusive” approaches (109) warrant investigation in the perinatal period.

Implementing mental health assessment for partners into clinical practice depends on acceptability to both health professionals and parents. Evidence regarding the acceptability of specific measures is limited but resonated with literature on acceptability in women, e.g., timing of administration, time required, clarity of wording (110). Here, fathers in the included studies reported mixed views, characterized by ambivalence; this echoes findings of a meta-synthesis of 20 studies that examined the broader support needs of partners of women with perinatal mental health disorders (50). Health professional views varied greatly, with some indication of variation by the culture of the profession, as well as the culture where the study was conducted, and that this may change over time. These findings resonate with reports of fathers' marginalization in services and this being linked to institutional and professional biases, including gender bias—often unconscious—against men as caregivers (111, 112). It consequently appears that while literature demonstrates prevalence and impact of partners' perinatal mental health difficulties, there are fundamental challenges to overcome in implementing effective assessment.

Fathers and health professionals identified possible challenges that were categorized across different levels: individual, practitioner, and service. Shared concerns in both groups included limited contact and its associated practical barriers (34, 49, 80, 82, 84, 86–89, 91), and resource implications (34, 49, 83, 87, 92), including the potential to compromise support offered to women. Health professionals expressed additional concerns regarding their knowledge, skills and confidence (80, 84, 87, 89–91), the lack of appropriate measures (85–88) and availability of onward referral routes (92, 95). Consideration of these findings alongside acceptability evidence concerning assessment of maternal mental health illustrates that many of the debates relevant to the introduction of universal routine perinatal mental health assessment of women, such as those outlined by the Marcé Society (26) apply here; some are amplified.

Women's reported barriers to help-seeking and accessing services for their mental health in the perinatal period include their ability to recognize their symptoms, stigma and self-blame, perceived purpose of assessment, perceived relevance to services, and health professional communication skills (101, 104, 110, 113). All of these are evident in the current review as relevant for fathers; moreover, they may be heightened, for example perceived relevance and stigma. Similarly, established barriers amongst health professionals regarding maternal mental health assessment are evident here; including, challenges at the practitioner level (e.g., knowledge, skills, confidence, attitude and scope of practice, fear of causing offense) and at the service level (e.g., lack of onwards referral options, resources/workload issues (time pressures), and tools being unavailable in different languages) (102–104, 110). It seems likely that these barriers relating to mental health assessment will be greater where co-parents are not themselves the intended recipients of services and that acceptability may vary with practice setting.

All studies examining acceptability in a practice setting concerned postnatal environments. This included universal assessment of fathers in the context of health visiting (public health nursing) services, and assessment in specialist settings where fathers may be more vulnerable to perinatal mental health disorders; specifically, where the child has health complications (e.g., NICU) or where support is needed around early parenting difficulties (e.g., early parenting services). There is some initial indication of acceptability when assessing fathers' depression symptoms in universal postnatal services and specialist early parenting services, and when assessing fathers' depression and trauma symptoms in intensive care settings. However, findings have been varied and studies have to-date focused on uptake rates and caution is needed in interpreting these behavioral measures as indicators of acceptability (46).

No practice-focused studies examined maternity services or specialist perinatal mental health services and it is plausible that views of professionals may vary in such services, where the “index patient” is the gestational parent, compared with services where the focus is the child. In addition, there may be additional challenges not captured here, for example the ability to document responses and onward referrals. It is also relevant that no studies examined the acceptability of *targeted* assessment on the basis of characteristics within the family (e.g., the mother's mental health, or the co-parent's mental health history). In such populations, prevalence of mental health disorders will be higher because parents' mental health is correlated (4) and because mental health history is known to increase likelihood of perinatal depression in fathers (114). This will have implications for the performance of the test (because the positive predictive value is directly linked to prevalence). In addition, parents and professionals may have different perceptions regarding the potential benefit of assessment, and there may be different opportunities for contact.

Any screening programme has the potential to do harm as well as good (115). In the context of maternal mental health assessment, a key ethical concern has been the introduction of routine assessment without appropriate onward pathways (26). This review found no studies evaluating the effectiveness of partners' mental health assessment although with the practice-based studies, there were occasional comments regarding uptake of support following assessment (81, 94) indicating the need to also address barriers to onward service use. No evidence was identified regarding effectiveness of assessment undertaken as part of a care pathway. Similarly, this review found no evidence that examined potential harm linked to assessment in partners. Some fathers themselves expressed concerns about the potential for their assessment to compete with women's support, and the wider literature on maternal mental health assessment has similarly noted that whilst some women welcome the presence of their partner or another family member for their mental health assessment, some professionals and women express concerns about this (57, 110, 116). This review identified no evidence on the acceptability of assessing couples' mental health together however some health professionals voiced concerns about potential tensions in working closely—albeit separately—with both parents, including knowledge or suspicion of inter-partner violence and domestic abuse.

Bringing together findings from the accuracy and acceptability studies, there is still much to be learned about the best way to introduce mental health assessment of fathers, other co-parents and partners. Relevant for policy and practice is the need to consider how this may vary if assessing on a universal basis (i.e., all partners) or targeted to groups considered higher risk (e.g., based on the mother's mental health). The accuracy studies have largely been conducted in the context of research and it has been argued elsewhere in relation to maternal mental health assessment that barriers to disclosure will be different and likely greater when tools are evaluated in practice contexts, influencing tools' psychometric properties (117). In light of the acceptability findings in the current evidence synthesis, it seems plausible that the context of disclosure will similarly be relevant for fathers and indeed that the gaps between accuracy in research and practice may be greater still.

Further research is needed to assess accuracy and acceptability in a range of practice settings, including antenatal clinics, health visiting and specialist perinatal mental health services, and with a range of stakeholders, including health professionals, co-parents and partners. It is strongly encouraged that future research not be limited to depression. Under-represented voices need to be actively sought to address the visibility of minority groups including minority ethnic parents, non-resident parents, step-parents, LGBT+ parents and other partners. Such research needs to examine challenges at the individual-, practitioner- and service-level and ethical considerations, including safeguarding, confidentiality, data protection, and the ability to adequately address identified risk. Relevant here is that depression-focused tools may themselves provide a marker for other disorders (56), necessitating a comprehensive approach to risk protocols and onward referrals. To avoid the challenges encountered when routine maternal mental health assessment was introduced in the UK and elsewhere, consideration is needed of care pathways, shifting from an emphasis on assessment and considering resource implications for each step. This includes practitioners' and services' abilities to document and act on identified risk. Here, there are opportunities for services to be evidence-generating, evaluating effectiveness by capturing care pathways and with attention to mental health and relationship outcomes for partners, for women and for children, and with economic data.

### Strengths and Limitations

Locating studies on diagnostic test accuracy and determining their eligibility was straightforward. In contrast, studies on acceptability were less easy to identify from the title and abstract, requiring a broader search strategy and further assessment at the level of full text. Although citation chaining was used to increase the likelihood of identifying relevant literature, it remains a possibility that some has been missed. Decisions regarding inclusion of potentially relevant literature were made by two reviewers, to promote the robustness of decision-making. In addition, to make the review manageable and promote transparency, it was intentional not to include papers where the only mention of acceptability concerned dropout or recruitment of fathers or other partners, for example in literature concerning correlates or prevalence of perinatal mental health. The broader literature on men's wider needs in the perinatal period and the extensive literature on women's acceptability of mental health assessment were also ineligible unless also discussing acceptability of partners' mental health assessment and it is recognized that there may be learning from these; for example, concerning assessment in the presence of a partner.

## Conclusion

Any parent or partner can experience perinatal mental health difficulties and partners of mothers who are experiencing perinatal mental health disorders may be particularly vulnerable to mental illness. Despite a small number of studies suggesting the accuracy and acceptability of screening tools with fathers in the postnatal period, this mixed methods evidence synthesis found that overall, there is not currently enough published evidence to indicate that using a specific tool, either on a universal basis or targeted to those in high-risk groups, would be accurate, acceptable and ultimately effective at identifying the mental health needs of partners and improving outcomes. The best available evidence concerns the EPDS however the results are highly varied. In addition, it has not been validated for use in the antenatal period. Some studies have found evidence indicating it may be feasible to use the EPDS in postnatal settings however parents' and professionals' perspectives demonstrate the challenges that exist at the individual-, practitioner- and service-level concerning assessment.

Understanding these challenges is vital for future implementation and evaluation. Even if we are not yet in a position to routinely introduce evidence-based assessment, professionals need to be alert to partners' mental health needs and able to respond. Services introducing assessment will need to devise systems for recording information on partners' mental health, with consideration of their responsibilities regarding different family members. Training and supervision can be used to help practitioners address gender bias and build confidence in working with partners. There is an urgent need for further research that is sensitive to practice settings and addresses concerns regarding possible harm, with assessment examined as part of a pathway. It is also essential that, as services begin to introduce assessment into practice, they collect good quality data that can contribute to ongoing service development and improvement, and attend to issues of inclusivity and equity of access.

## Data Availability Statement

The original contributions presented in the study are included in the article/Supplementary Material, further inquiries can be directed to the corresponding author/s.

## Author Contributions

ZD was the lead reviewer for all review elements and drafted the paper. ZD and VS led the design of the review. Data extraction was undertaken by ZD, VS, and JD. ZD led the synthesis process, with the synthesis refined by team discussions (all authors). All authors were involved with screening records for inclusion based on the title and abstract, with ZD and JD responsible for deciding which full-text articles were included in the review. All authors collaborated in writing and editing the paper.

## Conflict of Interest

The authors declare that the research was conducted in the absence of any commercial or financial relationships that could be construed as a potential conflict of interest.
