# Supplementary material for: Assessing the Mental Health of Fathers, Other Co-parents, and Partners in the Perinatal Period: Mixed Methods Evidence Synthesis
Source: Front Psychiatry. 2021 Jan 12;11:585479. doi: 10.3389/fpsyt.2020.585479 (PMC7835428; doi:10.3389/fpsyt.2020.585479)
Supplement: Supplementary file 1 [file Table_1.docx]

**Supplementary Material Table 1: Characteristics of included studies assessing diagnostic test accuracy in fathers (n=7)**

| **Publication/ Country/ Aims** | **Mental health disorder / Timing** | **Recruitment and selection bias** | **Sample details** | **Assessment** | **Flow and timing** | **Other details, including limitations** |
| --- | --- | --- | --- | --- | --- | --- |
| Areias et al. (1996)  Portugal  **Aims**  - Validation of EPDS in Portuguese mothers  - Comparative incidence of depression in mothers and fathers during pregnancy and after childbirth | Depression (type unspecified)  ***Time-point***  Pooled longitudinal data from 3 timepoints: antenatal (6 months) and postnatal (3, 12 months) | *Recruitment*  Mothers ≤24 weeks gestation recruited from antenatal clinics, inviting mothers; ‘husbands/partners’ were then invited (details N/R)  *Selection bias**  Consecutive sampling of women attending clinics. Of 80 mothers invited to participate, 65 consented. 54 ‘remained’ in the study (4 dropped out between timepoints; 2 had stillbirths; 5 moved or were uncontactable between 3 months and 12 months). 42 ‘husbands/ partners’ of the 54 mothers that remained in the study agreed to take part.  *Eligibility*  - Basic education to complete questionnaires | *Sample size*  - 42 fathers (and 54 mothers)  *Socio-demographics*  - Mean age 26.2 years (range 20-37)  - Majority employed (n=39)  - SES: range reported (Graffar Scale Class 1: 2%, 2: 13%, 3: 44%, 4: 39%, 5: 2%; categories 4-5 broadly contain working class families and part of category 3 contains manual workers).  *Paternal mental health history*  - Depression (n=9)  - GAD (n=2)  - Alcoholism (n= 1)  - Drug dependence (n=1) | **Index test (version)**  EPDS (Portuguese)  **Reference**  Schedule for Affective Disorders, regular and lifetime versions  **Administration**  *Mode and setting*  Index test self-completed; same setting used for index and reference (health setting at timepoints 1-2; home setting at timepoint 3)  *Blinding**  N/R  *Assessors*  1 appropriately trained psychologist | *Verification bias** Interviewed all at timepoint 1 and 3; unclear why a subsample (12/42) was used at timepoint 2 or how they were sampled  *Time interval**  Same day; order of administration unclear  *Analysis**  Various details missing; data before dropout appears not to have been used; paper reports cases at different timepoints but all data are pooled for analysis of performance and therefore could contain 2-3 datapoints from each participant | Authors noted their SADS criteria for depression may have been applied ‘less stringently’ given higher than anticipated numbers identified (both in men and women) but also noted high psychiatric history in sample.  Reports statistics for different ‘EPDS cut-offs’ without specifying, e.g. cut-off of 8 could mean ≥7 or ≥8.  Authors note that drop out from the longitudinal study was higher for fathers (numbers N/R; parents’ views were not assessed). |
| Ballard et al. (1994)  UK  **Aims**  - Comparative prevalence of depression in mothers and fathers at 6 weeks and 6 months postpartum  - Concurrent depression in couples | Depression (type unspecified)  ***Time-point***  Postnatal (6 months) | *Recruitment*  Recruited from postnatal maternity wards, using an ‘unselected postnatal sample’ – all eligible mothers were approached on days the researcher visited the ward; unclear how fathers were then invited; explanatory letter gave instructions about both partners completing separately  *Selection bias**  Consecutive sampling of women present when researcher attending. 224 mothers were approached 🡪 6 excluded on basis of language; 15 declined; 3 unavailable (babies on SCBU) 🡪 200 mothers entered the studies 🡪 178 couples at 6 weeks postnatal follow-up (further 12 singles completed but N/R whether mother/father; no diagnostic interview completed at 6 weeks) 🡪 148 couples completed index test at 6 months postnatal (further 6 from singles but N/R mother/father) ; weekly reminders sent and follow up by telephone if no response 🡪 48 fathers had diagnostic interview  *Eligibility*  - Married or cohabiting  - Excluded if they did not ‘speak adequate English’ | *Sample size*  - 178 fathers (and 178 mothers) at 6 weeks resulting in 48 fathers at interview  *Socio-demographics (original sample)*  - Mean age 27.7 (SD N/R; original sample)  - SES: majority ‘social classes III manual and IV’ based on paternal occupation; mean Jarman score (deprivation) 20.9 (SD 19.3) | **Index test (version)**  EPDS (English) (early version, comprising 13 items)  **Reference**  Psychiatric Assessment Scale  **Administration**  *Mode and setting*  Index test self-completed at home and returned by post; interview setting N/R  *Blinding**  Yes  *Assessors*  2 appropriately trained interviewers completed interviews; inter-rater reliability reported as ‘satisfactory’ | *Verification bias** Couple invited for interview on basis of mother’s score: all ≥13 on the EPDS, 50% of those scoring 10-12, and 10% scoring <10  *Time interval**  Interviews were set within 2 weeks of the date the completed questionnaire was received  *Analysis**  Chose the optimal cut-off point based on data reported in the original Master’s dissertation as offering optimal specificity and sensitivity | Used 13-item version, on basis that it appears to have better sensitivity. This was an early version of the EPDS where scores are likely to be higher, due to more items.  Diagnostic interviews only conducted at 6 months, not 6 weeks.  The original Master’s dissertation (Ballard, 1992) was unavailable but reports full statistics across EPDS cut-off scores and also validated the GHQ, reporting that the EPDS performed better than the GHQ.  Authors noted a strength of the study was that it was unrestricted by age, social status or perinatal complications |
| Edmondson et al. (2010)  UK  **Aims**  - Determine whether a reliable EPDS cut-point can be established to screen fathers, by examining its validity against a structured clinical interview | Major depression / generalized anxiety disorder (GAD)  ***Time-point***  Postnatal (7-14 weeks) | *Recruitment*  Recruited from postnatal maternity wards either directly (if present) or via the mother  *Selection bias**  Unclear whether consecutive sampling of all parents on postnatal wards. 4,107 men were sent questionnaire at 7 weeks postnatal; 38.0% (1,562) returned; reminders were sent to those that did not reply within 2 weeks 🡪 invited 340 couples to interviews 🡪 192 couples agree to home visit 🡪 complete data available for 189 fathers (and 184 mothers)  *Eligibility*  - Sufficient English to complete questionnaires | *Sample size*  189 fathers (and 184 mothers)  *Socio-demographics*  - Mean paternal age 35.0 years (SD 5.86)  - 59.4% first time parents (not presented by father/mother)  - 191/192 couples were married/living together  *Paternal mental health history*  - Depression (n=35; 18.4%) | **Index test (version)**  EPDS (English)  **Reference**  Structured Clinical Interview for DSM-IV (SCID), modules for depression and anxiety disorders  **Administration**  *Mode and setting*  Index test self-completed at home and returned by post; interviews conducted at home  *Blinding**  N/R  *Assessors*  Psychologists and psychiatrists trained in the interview (SCID) | *Verification bias**  Invited all fathers with scores of ≥10 and a random sample of 1 in 4 of those fathers with scores <10 to interview  *Time interval**  Mean interval of 4.8 weeks (SD 3.04), with interviews approximately 14 weeks postnatal  *Analysis**  Weighted statistics were estimated for whole study population, due to over-representing high-scoring fathers – but only for some of the analyses. Weighted by re-running for a modelled dataset with 4 times the number of low-scoring fathers to approximate the original sample population. | Reported that older men were more likely to participate.  “The relatively modest participation rate means the results may not be fully generalisable to the whole population.” (p.365)  Authors recommended future studies aim to deliver instruments at the same assessment but also cited research with mothers that indicated such time intervals may not be problematic. |
| Lai et al. (2010)  Hong Kong  **Aims**  - Validate the Chinese EPDS, BDI, PHQ-9 in screening for postnatal depression among Chinese fathers in Hong Kong  - Compare the tools’ psychometric properties  - Estimate prevalence of depression at 8 weeks postpartum using structured clinical interview | Depression (minor/ major)  ***Time-point***  Postnatal (10 weeks) | *Recruitment*  Recruited from postnatal wards at a “University-affiliated general hospital”, recruiting partners of women consecutively admitted  *Selection bias**  Consecutive sampling. Approached 2,351 men and their partners with overall response rate of 44%; then sent measures by post at 8 weeks postnatal; if not returned within 3-4 weeks, reminder call was made; if not returned within further 2 weeks, contacted again 🡪 number completing index test unclear 🡪 . 551 fathers had diagnostic interview  *Eligibility*  - Limited to men of Chinese ethnicity with permanent residency rights in Hong Kong; noted need for separate study of immigrants | *Sample size*  551 fathers  *Socio-demographics*  -Mean paternal age 33.4 years (SD 5.9, range 18-59), -Mean number of children (including new child) 1.3 (SD 0.5, range 1-4)  -Mean monthly income (Hong Kong dollars) 37,493 (SD 85823)  - Range of social classes (classes I-V respectively 8.7, 26.1, 58.8, 3.3, 3.1%)  - Majority married (96.7%, 3.1% cohabiting, 0.2% single)  - Range of education (0.2% none, 0.2% kindergarten, 3.3% primary, 56.1% secondary, 40.3% University)  - Range of occupation categories (58.6% skilled and 35.0% professional/ semi-professional)  *Paternal mental health history*  - Past/current psychiatric history (10/548; 1.8%) | **Index test (version)**  EPDS, BDI, PHQ-9 (Chinese)  **Reference**  Structured Clinical Interview for DSM-IV, non-patient version (SCID-NP)  **Administration**  *Mode and setting*  Index tests self-completed at home and returned by post; interviews conducted at hospital  *Blinding**  Yes  *Assessors*  1 assessor; training unclear but works in psychiatry department | *Verification bias** Invited all scoring ≥11 on BDI or ≥10 on EPDS, plus 40% of “low scorers” chosen at random  *Time interval**  Mean interval of 7 days (mean 6.87, SD 4.87)  *Analysis**  Analysed all that completed interview, but did not conduct weighted analyses to explore verification bias | The hospital “serves over 1.5 million people of diverse socio-economic status”.  Authors note the interview is semi-structured and can be used in a “culturally informed way”, with interviewer able to ask about “indigenous idioms of distress” (p.82).  Appears to be a typographical error in Table 2 where BDI cut-off 10/11 should read 9/10, as all others are consecutive numbers.  Reported the response rate (44%) as being low but similar to other studies with men “suggesting that fathers in general are reluctant to complete self-report questionnaires” (p.84); participants expressed concern about the time involved in completing the (index) tools.    The authors noted the need for validation within the relevant culture and the range of scores across studies. |
| Massoudi et al. (2013)  Sweden  **Aims**  - Validate the Swedish EPDS in fathers relation to DSM-IV criteria for major and minor depression  - Investigate the factor structure of EPDS in fathers and mothers  - Examine if EPDS could be useful in detecting anxiety in fathers and compare it with HADS-A | Depression (minor/ major) / anxiety (type unspecified)  ***Time-point***  Postnatal (3-4 months) | *Recruitment*  Recruited by child health nurses at first home visit, within 10 days postnatal (when father usually on paternity leave); usually recruited couples together; if not, mother passed on to father, to read and give consent  *Selection bias**  Consecutive sampling of couples. 1,268 approached 🡪 1,014 consented (80%) 🡪 885 fathers (87%), 926 mothers (91%), 858 couples (85%) returned the postal questionnaire at 3 months postnatal); up to 2 reminders were sent to non-respondents 🡪 invited 337 to interview 🡪 262 fathers had diagnostic interview (65 could not be reached in the time period and 9 declined; those that did not take part did not differ on any of the characteristics assessed e.g. age, parity, occupation, education, mean scores on EPDS/HADS-A).  *Eligibility*  - Living together;  - Fluent enough in Swedish to understand tools and interview conducted in Swedish, and could fully understand the written and oral information | *Sample size*  262 fathers  *Socio-demographics*  Mean paternal age 33 years (20-51 range); 99.8% living with child’s mother  46% first-time fathers.  95% completed upper secondary school and 37% attended college or university  *Paternal mental health history*  N/R | **Index test (version)**  EPDS, HADS-A (Swedish)  **Reference**  Primary Care Evaluation of Mental Disorders (Prime-MD), modules for depression and anxiety disorders  **Administration**  *Mode and setting*  Index tests self-completed at home and retuned by post; interview conducted by telephone.  *Blinding**  Yes  *Assessors*  Experienced clinical psychologists; inter-rater reliability ensured through regular checks | *Verification bias**  Invited all scoring ≥10 on the EPDS, ≥4 on EPDS-3A, or ≥9 on the HAD anxiety scale, and a random sample of low-scoring (1 low-scoring father for every 2 high-scoring fathers, selected the 1^st^ low scoring postal questionnaire opened immediately after 2 high scoring)  *Time interval**  Majority within 2-3 weeks (71.5% within 2 weeks, 24% within 3 weeks, 4.5% within 4-6 weeks)  *Analysis**  Weighted statistics were estimated for whole study population, due to over-representing high-scoring fathers. | Non-participants did not differ regarding age, parity or occupation but there was a significantly highly number of fathers where native language was not English in non-participants (22% vs. 8%).  There appears to be inconsistency in numbers reported on p.70 (8/262 (3.1%) major depression and 20/262 (10.7%) minor depression) vs. p.71 (3.4% and 11%).  Authors note that conducting interviews by telephone may not be as accurate but may have helped facilitate participation.  Authors noted wide confidence intervals.  Study also examined EPDS-3A but did not report the results in full because the factor structure was not found in fathers and therefore only reported for EPDS. |
| Matthey et al. (2001)  Australia  **Aims**  - Validate the EPDS in fathers in relation to ‘distress caseness’  - Compare EPDS item endorsement in fathers and mothers | Depression (minor/ major), ‘distress’ (minor/ major depression, adjustment disorder with anxiety (all criteria for GAD except duration of 6 months), panic disorder, specific phobia)  ***Time-point***  Postnatal (6-7 weeks) | *Recruitment*  Recruited via antenatal classes, invited couples attending preparation for parenthood classes in evenings, paid for by couples (suggests issues of equity); unclear if consecutive recruitment of couples attending classes; part of a larger study on effectiveness of an intervention for postnatal distress  *Selection bias**  Unclear whether consecutive sampling of all couples attending classes. 251 couples recruited (number approached N/R) 🡪 sent questionnaires 6-7 weeks postnatal 🡪 218 fathers (and 238 mothers) participated  *Eligibility*  - Attending the antenatal classes | *Sample size*  218 fathers (and 238 mothers)  *Socio-demographics*  Mean paternal age 29.1 years (SD 4.6).  All married or ‘de facto’ relationship.  All first-time fathers.  Means years education 12.9 (SD N/R).  *Paternal mental health history*  N/R | **Index test (version)**  EPDS (English)  **Reference**  Diagnostic Interview Schedule  **Administration**  *Mode and setting*  Index tests self-completed at home and collected by researcher; interviews conducted at home  *Blinding**  Unclear; appears index was contained in sealed envelope and assessor therefore blinded; some may have completed after interview  *Assessors*  Trained research assistant, with reliability checks conducted | *Verification bias**  All fathers were invited (i.e. no criterion applied on basis of index scores)  *Time interval**  Unclear whether all completed on the same day, with varied order of administration, or if any completed 3 days prior to interview  *Analysis**  One further case of a father with major depression who had completed the EPDS but was not part of the study was added to the data to increase the number of cases.  Analysed all that completed interview, but did not conduct weighted analyses to explore verification bias | English versions assumed; all couples were recruited from paid-for preparation for parenthood classes and interpreters/ translation not mentioned.  CES-D also completed but results N/R.  Sample sizes vary for different analyses (200-218 fathers).  Drop out prior to interview was higher in fathers (34 vs. 13 mothers). Drop out was due to inability to contact or not at home on at least 3 occasions for pre-arranged interview. Three clinical interviews were stopped part-way through “due to the inability of the participant to continue (two with excessive tiredness, and one with apparent high alcohol intake)” (p.178)  Adjustment disorder with anxiety was used instead of GAD because GAD is defined as duration of ≥6 months, which was not possible at this timepoint. Panic disorder was assessed but not reported.  Authors note the validity of diagnostic interviews should not be assumed, including the potential for errors and that parents may not “always answer truthfully to questions about their mental health” (p.182), questioning whether false positives on the EPDS could actually be true cases. |
| Tran et al. (2012)  Vietnam  **Aims**  - Validate 3 instruments for screening perinatal non-psychotic common mental health disorders in men in Vietnam whose partners were pregnant or had recently given birth  - Estimate prevalence of these disorders in men | Non-psychotic common mental health disorders (including major depression, dysthymia, GAD, panic disorder)  ***Time-point***  Pooled data: spanning antenatal (~28 weeks) and postnatal (~6 weeks) | *Recruitment*  Recruited via community health workers - partners of participants in authors’ other studies were eligible to participate; study sites were randomly selected and all eligible women were invited. Health worker invited partner when accompanying to mother’s research interview, or at home visits associated with baby’s immunisation programme.  *Selection bias**  Consecutive sampling of women. 364/392 (93%) eligible women participated, including 360 who were partnered 🡪 231/360 partners (64%) agreed to participate. Main reasons for partners’ non-participation: work commitments / working away. Participation higher in urban (99/130, 76.2%) than rural (136/230, 59.1%).  *Eligibility*  - Women were ≥28 weeks pregnant or 4-6 weeks postnatal and registered for care at commune health station. | *Sample size*  231 fathers (and 360 mothers)  *Socio-demographics*  Paternal age 31 years (SD 6.3, range 20-49). 16.4% had not completed primary school. 79% generated income through agricultural or manual work. No significant differences between the women whose partners did and did not take part.  Breakdown of gestation and time post-birth N/R. Approximately 55% were partners of pregnant women (based on 199/364 women being pregnant).  *Paternal mental health history*  N/R | **Index test (version)**  EPDS, Zung’s self-rated anxiety scale (SAS), GHQ-12 (Vietnamese)  **Reference**  Structured Clinical Interview for DSM-IV (SCID), modules for depression, GAD and panic disorder  **Administration**  *Mode and setting*  Index tests administered as structured interview by health research workers; both completed in commune health station  *Blinding**  Yes  *Assessors*  1 psychiatrist | *Verification bias**  All fathers were invited (i.e. no criterion applied on basis of index scores)  *Time interval**  Same day; order of administration unclear  *Analysis**  Analysed all that completed interview, but did not conduct weighted analyses to explore verification bias | Note: low/middle-income setting  Authors note the interview was chosen because gold standard in ‘diverse cultural settings and countries’  Authors reported findings may not be representative of population because only open to those whose partners (the mothers) taking part in a related study.  Authors reported the cut-off scores to detect clinically significant symptoms are lower than those reported in high-income settings; however, this study also used a broad approach (anxiety or depression). Authors link this to sociocultural differences concerning emotion expression and that parents in some cultures may not have a “wide emotional vocabulary”, making questions about related but separate emotions confusing and difficult to respond to; in addition, tools may be insensitive to people living in poverty and sustained adversity, asking about symptoms as “difference to a usual state”.  Authors reported the measures were “acceptable and comprehensible to participants” (p.108) but no data is provided and there is no indication of capturing parents’ views. |

Note: * = QUADAS-2 criteria; CES-D = Centre for Epidemiological Studies-Depression Scale; EPDS = Edinburgh Postnatal Depression Scale; GHQ = General Health Questionnaire; HADS-A = anxiety subscale of the Hospital Anxiety and Depression Scale; N/R = not reported; PHQ = Patient Health Questionnaire; SCBU = special care baby unit; SD = standard deviation; SES = socio-economic status; Zung SAS = Zung's Self-rated Anxiety Scale
