# Supplementary material for: Assessing the Mental Health of Fathers, Other Co-parents, and Partners in the Perinatal Period: Mixed Methods Evidence Synthesis
Source: Front Psychiatry. 2021 Jan 12;11:585479. doi: 10.3389/fpsyt.2020.585479 (PMC7835428; doi:10.3389/fpsyt.2020.585479)
Supplement: Supplementary file 2 [file Table_2.DOCX]

**Supplementary Material Table 2: Summary of risk of bias of included studies assessing diagnostic test accuracy in fathers (n=7), following QUADAS-2 criteria**

| **Publication/ Country** | **Participant selection** | **Index test** | **Reference standard** | **Flow and timing** |
| --- | --- | --- | --- | --- |
| Areias et al. (1996)  Portugal | *Sampling*  Consecutive (low risk)  *Dropouts*  Yes (high risk) | *Blinding*  Not reported (risk unclear)  *Order of administration*  Unclear (risk unclear) | *Reference standard likely to correctly classify*  Yes (low risk) | *Time interval*  Same day (low risk)  *Verification bias*  Subsample received reference standard; unclear if this was dependent on the index test (risk unclear)  *Analysis*  Multiple datapoints from each person and all data pooled (high risk) |
| Ballard et al. (1994)  UK | *Sampling*  Consecutive (low risk)  *Dropouts*  Yes (high risk) | *Blinding*  Yes (low risk)  *Order of administration*  Index completed first (low risk) | *Reference standard likely to correctly classify*  Yes (low risk) | *Time interval*  Within 2 weeks (medium risk)  *Verification bias*  Subsample received reference standard; sampled on basis of mother’s score on the index test (high risk)  *Analysis*  Analysed all that completed both, but did not conduct weighted analyses (medium risk) |
| Edmondson et al. (2010)  UK | *Sampling*  Unclear whether consecutive (risk unclear)  *Dropouts*  Yes (high risk) | *Blinding*  Not reported (risk unclear)  *Order of administration*  Index completed first (low risk) | *Reference standard likely to correctly classify*  Yes (low risk) | *Time interval*  Mean interval of 4.8 weeks (SD 3.04) (high risk)  *Verification bias*  Subsample received reference standard; sampled on basis of index test score (high risk)  *Analysis*  Weighted statistics available for some of the analyses (low risk) |
| Lai et al. (2010)  Hong Kong | *Sampling*  Consecutive (low risk)  *Dropouts*  Yes (high risk) | *Blinding*  Yes (low risk)  *Order of administration*  Index completed first (low risk) | *Reference standard likely to correctly classify*  Yes (low risk) | *Time interval*  Mean interval of 7 days (mean 6.87, SD 4.87) (medium risk)  *Verification bias*  Subsample received reference standard; sampled on basis of index test score (high risk)  *Analysis*  Analysed all that completed both, but did not conduct weighted analyses (medium risk) |
| Massoudi et al. (2013)  Sweden | *Sampling*  Consecutive (low risk)  *Dropouts*  Yes (high risk) | *Blinding*  Yes (low risk)  *Order of administration*  Index completed first (low risk) | *Reference standard likely to correctly classify*  Yes (low risk) | *Time interval*  Majority within 2-3 weeks (medium risk)  *Verification bias*  Subsample received reference standard; sampled on basis of index test score (high risk)  *Analysis*  Weighted statistics were available for all analyses (low risk) |
| Matthey et al. (2001)  Australia | *Sampling*  Unclear whether consecutive (risk unclear)  *Dropouts*  Yes (high risk) | *Blinding*  Not reported (risk unclear)  *Order of administration*  Unclear (risk unclear) | *Reference standard likely to correctly classify*  Yes (low risk) | *Time interval*  Unclear but within 3 days (low risk)  *Verification bias*  All fathers were invited (i.e. no criterion applied on basis of index scores) (low risk)  *Analysis*  Analysed all that completed both and an additional father that was not part of the study, but did not conduct weighted analyses (medium risk) |
| Tran et al. (2012)  Vietnam | *Sampling*  Consecutive (low risk)  *Dropouts*  Yes (high risk) | *Blinding*  Yes (low risk)  *Order of administration*  Unclear (risk unclear) | *Reference standard likely to correctly classify*  Yes (low risk) | *Time interval*  Same day (low risk)  *Verification bias*  All fathers were invited (i.e. no criterion applied on basis of index scores) (low risk)  *Analysis*  Analysed all that completed both, but did not conduct weighted analyses (medium risk) |
