# Supplementary material for: Assessing the Mental Health of Fathers, Other Co-parents, and Partners in the Perinatal Period: Mixed Methods Evidence Synthesis
Source: Front Psychiatry. 2021 Jan 12;11:585479. doi: 10.3389/fpsyt.2020.585479 (PMC7835428; doi:10.3389/fpsyt.2020.585479)
Supplement: Supplementary file 3 [file Table_3.DOCX]

**Supplementary Material Table 3: Results and recommendations of included studies assessing diagnostic test accuracy in fathers (n=7)**

| **Publication/ Country** | **Assessment** | **Mental health disorder; cases n(%)** | **Cut-point** | **Sens**  **(%)** | **Spec**  **(%)** | **PPV (%)** | **NPV (%)** | **Mis. (%)** | **Other results** | **Authors’ conclusions and recommendations** |
| --- | --- | --- | --- | --- | --- | --- | --- | --- | --- | --- |
| Areias et al. (1996)  Portugal | **Index test (version)**  EPDS (Portuguese)  **Reference**  Schedule for Affective Disorders, regular and lifetime versions  **Timing**  Pooled longitudinal data: antenatal (6 months) and postnatal (3, 12 months) | Depression (type unspecified)  12/96 (12.5) | 8 | 40 | 93 | 67 | 82 |  | *AUC*  N/R  *Item endorse.*  N/R | Authors do not specify whether an “EPDS cut-off of 8” means >8 (greater than 8) or ≥ 8 (ie. 8 or more).  Authors report that poor sensitivity (i.e. under-identification) was a concern when used with fathers, whereas both specificity and sensitivity were high with mothers. Authors do not nominate a cut-off point that offers the best performance, for either group however the scores presented indicate a cut-off of 8 provides the best balance of sensitive and specificity.  Authors report EPDS able to “discriminate successfully” between minor and major depression in mothers but not fathers. |
|  |  |  | 9 | 40 | 81 | 36 | 83 |  |  |  |
|  |  |  | 10 | 40 | 81 | 36 | 83 |  |  |  |
|  |  |  | 11 | 20 | 86 | 29 | 79 |  |  |  |
|  |  |  | 12 | 20 | 92 | 40 | 80 |  |  |  |
|  |  |  | 13 | 10 | 94 | 33 | 79 |  |  |  |
| Ballard et al. (1994)  UK | **Index test (version)**  EPDS (13-item; English)  **Reference**  Psychiatric Assessment Scale  **Timing**  Postnatal (6 months) | Depression (type unspecified)  6/48 (12.5) | ≥13 | 85.7 | 75.0 | N/R | N/R |  | *AUC*  N/R  *Item endorse.*  N/R | Original Master’s thesis reports details for other cut-offs but this was unavailable.  Discussion notes that mothers had significantly more symptoms than fathers (although symptoms themselves similar) and suggest that fathers may have less severe depressive illness or may be less likely to report. At the same cut-off, sensitivity was 95.7% for mothers and specificity was 71.0%.  Concludes tool is suitable for use in a two-stage design.  Authors argued the need to develop “effective screening and treatment methods” for “highly vulnerable families” (depressed female partners, unemployed, unsupportive relationship/partner). |
| **Publication/ Country** | **Assessment** | **Mental health disorder; cases n(%)** | **Cut-point** | **Sens**  **(%)** | **Spec**  **(%)** | **PPV (%)** | **NPV (%)** | **Mis. (%)** | **Other results** | **Authors’ conclusions and recommendations** |
| Edmondson et al. (2010)  UK | **Index test (version)**  EPDS (English)  **Reference**  Structured Clinical Interview for DSM-IV (SCID), modules for depression and anxiety disorders  **Timing**  Postnatal (7-14 weeks) | Depression (major)  19/189 (10.0) | ≥7 | 100.0 | 52.9 | 19.2 | 100.0 | 42.3 | *AUC*  0.916 (CI 0.864-0.967)  *Item endorse.*  N/R | The EPDS has “reasonable sensitivity and specificity” (p.367).  “The study shows it is possible to screen fathers for depression in the postnatal period and it may be valuable to administer this measure to new fathers.” (p.365).  Score of ≥11 chosen as optimal because a high level of sensitivity is need for a “screening test for depression” (p.367).  No recommendation is made about screening for caseness using a broader approach that includes anxiety. |
|  |  |  | ≥8 | 100.0 | 60.0 | 21.8 | 100.0 | 36.0 |  |  |
|  |  |  | ≥9 | 100.0 | 65.3 | 24.4 | 100.0 | 31.2 |  |  |
|  |  |  | ≥10 | 94.7 | 68.2 | 25.0 | 99.1 | 29.1 |  |  |
|  |  |  | ≥11* | 89.5 | 78.2 | 31.5 | 98.5 | 20.6 |  |  |
|  |  |  | ≥12 | 78.9 | 84.7 | 36.6 | 97.3 | 15.9 |  |  |
|  |  |  | ≥13 | 68.4 | 90.6 | 44.9 | 96.2 | 11.7 |  |  |
|  |  |  | ≥14 | 63.2 | 94.1 | 54.5 | 95.8 | 9.0 |  |  |
|  |  |  | ≥15 | 52.6 | 96.5 | 62.5 | 94.8 | 5.2 |  |  |
|  |  |  | ≥11*^W^ | 77.3 | 92.9 |  |  | N/R |  |  |
|  |  | Depression (major) / generalized anxiety disorder (GAD)  26/189 (13.8) | ≥9* | 92.0 | 66.5 | N/R | N/R | N/R |  |  |
| **Publication/ Country** | **Assessment** | **Mental health disorder; cases n(%)** | **Cut-point** | **Sens**  **(%)** | **Spec**  **(%)** | **PPV (%)** | **NPV (%)** | **Mis. (%)** | **Other results** | **Authors’ conclusions and recommendations** |
| Lai et al. (2010)  Hong Kong | **Index test (version)**  EPDS, BDI, PHQ-9 (Chinese)  **Reference**  Structured Clinical Interview for DSM-IV, non-patient version (SCID-NP)  **Timing**  Postnatal (10 weeks) | Depression (minor/ major)  17/551 (3.1) | EPDS ≥8 | 100 | 89 | 31 | 100 |  | *AUC*  EPDS 0.97 (CI 0.95-0.99)  BDI 0.93 (CI 0.88-0.97)  PHQ-9 0.92 (CI 0.86-0.98)  The EPDS was more accurate than BDI (p=0.02) and PHQ-9 (p=0.04).  *Item endorse.*  All depressed men reported undue self-blaming and feeling over-whelmed; half reporting thought of self-harming (vs. only 5% in non-depressed) | Recommends “using the EPDS to screen for postnatal depression in Chinese men” (p.83), also noting that he EPDS is easy and quick to complete, and can be used free of charge.  “EPDS was significantly more accurate than the BDI and the PHQ-9 in detecting postnatal depression among Chinese men….It could be applied as a supplementary assessment tool in Chinese fathers who may be reluctant to disclose their depressive symptoms in face-to-face interviews.” (p.80); however, in this study all tools were completed face-to-face, administered by health research workers.  “Cases of depression may go underreported if using face-to-face interviews as men may be reluctant to disclose their depressive symptoms. Healthcare professionals working with Chinese ethnic groups may consider using the EPDS as a supplementary assessment tool in identifying men at risk of depression. It may serve as an entry point for engaging individuals….in clinical assessment and intervention” (p.84). |
|  |  |  | ≥9 | 91 | 92 | 37 | 99 |  |  |  |
|  |  |  | ≥10 | 91 | 94 | 42 | 99 |  |  |  |
|  |  |  | ≥11* | 91 | 97 | 57 | 99 |  |  |  |
|  |  |  | ≥12 | 66 | 98 | 65 | 98 |  |  |  |
|  |  |  | ≥13 | 41 | 98 | 60 | 96 |  |  |  |
|  |  |  | ≥14 | 35 | 98 | 62 | 96 |  |  |  |
|  |  |  | BDI ≥4 | 100 | 71 | 16 | 100 |  |  |  |
|  |  |  | ≥5 | 100 | 76 | 19 | 100 |  |  |  |
|  |  |  | ≥6* | 100 | 81 | 21 | 100 |  |  |  |
|  |  |  | ≥7 | 90 | 84 | 23 | 99 |  |  |  |
|  |  |  | ≥8 | 82 | 85 | 23 | 99 |  |  |  |
|  |  |  | ≥9 | 67 | 89 | 26 | 98 |  |  |  |
|  |  |  | ≥10 | 64 | 93 | 38 | 97 |  |  |  |
|  |  |  | PHQ-9 ≥2 | 100 | 62 | 14 | 100 |  |  |  |
|  |  |  | ≥3 | 93 | 69 | 17 | 83 |  |  |  |
|  |  |  | ≥4* | 85 | 81 | 23 | 98 |  |  |  |
|  |  |  | ≥5 | 77 | 87 | 27 | 98 |  |  |  |
|  |  |  | ≥6 | 71 | 91 | 35 | 98 |  |  |  |
|  |  |  | ≥7 | 70 | 94 | 43 | 98 |  |  |  |
|  |  |  | ≥8 | 56 | 96 | 48 | 97 |  |  |  |
| **Publication/ Country** | **Assessment** | **Mental health disorder; cases n(%)** | **Cut-point** | **Sens**  **(%)** | **Spec**  **(%)** | **PPV (%)** | **NPV (%)** | **Mis. (%)** | **Other results** | **Authors’ conclusions and recommendations** |
| Massoudi et al. (2013)  Sweden | **Index test (version)**  EPDS, HADS-A (Swedish)  **Reference**  Primary Care Evaluation of Mental Disorders (Prime-MD), modules for depression and anxiety disorders  **Timing**  Postnatal (3-4 months) | Depression (major)  8/262 (3.1) | ≥10 | 100.0 | 72.7 | 10.3 | 100.0 |  | *AUC*  N/R  *Item endorse.*  On the EPDS, feeling overwhelmed was endorsed by >70% fathers; self-harm was excluded from factor structure analyses as endorsed by only 3% of fathers and mothers.  *Other*  Paper also reports for all cut-offs the CI for sens/spec and LR+/LR-. | Authors recommend assessment be targeted, not universal; reporting that although all fathers with major depression would be identified using the EPDS, four in five fathers would be false positives. Selective assessment with the EPDS is recommended “when signs of distress or difficulties are noted, or in case where the partner [mother] is depressed.” (p.73).  For major depression, there is high sensitivity and specificity but low PPV; for minor depression, accuracy is only modest, questioning if the EPDS should be used to screen for minor depression. Concludes that neither the EPDS-3A or HADS-A should be used to screen for anxiety due to not reaching “acceptable validity”.  Authors propose that the relatively high cut-off for fathers found here (compared with the wider literature) may be due to there being “no major difference” in how men and women express symptoms of major depression in Sweden, whereas minor depression may seem “more legitimate” for mothers; they also speculate that this may be the reason for poor accuracy for minor depression.  Recommends developing measures that are “more sensitive to the underlying features of distress in new fathers” (p.73). Also notes that signs of distress or difficulties are often not seen by health professionals and therefore encourages more involvement of fathers or a more structured approach to appointments. |
|  |  |  | ≥11 | 100.0 | 83.9 | 16.3 | 100.0 |  |  |  |
|  |  |  | ≥12* | 100.0 | 87.4 | 20.0 | 100.0 |  |  |  |
|  |  |  | ≥13 | 100.0 | 91.3 | 21.4 | 99.1 |  |  |  |
|  |  |  | ≥10^W^ | 100.0 | 89.4 |  |  |  |  |  |
|  |  |  | ≥11^W^ | 100.0 | 93.0 |  |  |  |  |  |
|  |  |  | ≥12*^W^ | 100.0 | 94.9 |  |  |  |  |  |
|  |  |  | ≥13^W^ | 51.7 | 96.7 |  |  |  |  |  |
|  |  | Depression (minor/ major)  28/262 (10.7) | ≥8 | 85.7 | 58.1 | 19.7 | 97.1 |  |  |  |
|  |  |  | ≥9* | 85.7 | 67.1 | 23.8 | 97.5 |  |  |  |
|  |  |  | ≥10 | 75.0 | 76.0 | 27.3 | 96.2 |  |  |  |
|  |  |  | ≥11 | 57.1 | 85.9 | 32.7 | 94.4 |  |  |  |
|  |  |  | ≥8^W^ | 65.3 | 81.6 | 19.7 | 97.1 |  |  |  |
|  |  |  | ≥9^*W^ | 66.0 | 86.3 | 23.8 | 97.5 |  |  |  |
|  |  |  | ≥10^W^ | 49.0 | 90.8 | 27.3 | 96.2 |  |  |  |
|  |  |  | ≥11^W^ | 34.3 | 93.9 | 32.7 | 94.4 |  |  |  |
|  |  | Anxiety (type unspecified)  29/262 (11.1) | EPDS ≥7 | 89.7 | 49.4 | 18.1 | 97.5 |  |  |  |
|  |  |  | ≥8* | 86.2 | 58.4 | 20.5 | 97.1 |  |  |  |
|  |  |  | ≥9 | 58.6 | 63.9 | 16.8 | 92.5 |  |  |  |
|  |  |  | EPDS ≥7^W^ | 74.1 | 74.7 |  |  |  |  |  |
|  |  |  | ≥8^*W^ | 66.2 | 81.8 |  |  |  |  |  |
|  |  |  | ≥9^W^ | 31.5 | 84.6 |  |  |  |  |  |
|  |  |  | HADS-A ≥6 | 70.0 | 42.7 | 13.6 | 91.7 |  |  |  |
|  |  |  | ≥7 | 55.2 | 50.9 | 12.4 | 90.0 |  |  |  |
|  |  |  | ≥8* | 50.0 | 60.4 | 13.3 | 90.8 |  |  |  |
|  |  |  | HADS-A ≥6^W^ | 45.0 | 67.9 |  |  |  |  |  |
|  |  |  | ≥7^W^ | 28.4 | 76.3 |  |  |  |  |  |
|  |  |  | ≥8*^W^ | 23.3 | 83.4 |  |  |  |  |  |
| **Publication/ Country** | **Assessment** | **Mental health disorder; cases n(%)** | **Cut-point** | **Sens**  **(%)** | **Spec**  **(%)** | **PPV (%)** | **NPV (%)** | **Mis. (%)** | **Other results** | **Authors’ conclusions and recommendations** |
| Matthey et al. (2001)  Australia | **Index test (version)**  EPDS (English)  **Reference**  Diagnostic Interview Schedule  **Timing**  Postnatal (6-7 weeks) | Depression (minor/ major)  7/200 (3.5) | ≥3 | 100.0 | 37.8 | 5.5 | 100.0 | 60.0 | *AUC*  N/R  *Item endorse.*  No gendered differences in endorse-ment for self-blame, sleep difficulties, or self-harm; for remaining items, men had lower endorse-ment and this was statistically significant for crying | Authors (p.182) recommend using the EPDS routinely in all new fathers and using a cut-off of 5/6 (i.e. 6 or more) to “screen for both depressive and anxiety disorder” and note the optimal cut-off for mothers was 7/8 (i.e. 8 or more).  Authors argue the need to screen not just for depression but for distress; they note it is unknown “whether new fathers are more likely to experience distress through anxiety symptoms, or whether they are more prepared to admit such symptoms rather than depressive symptoms” (p.182). Authors therefore argue “the importance of not just screening for depression” in new fathers (p.182). The lower cut-off minimizes ‘missing’ any cases but increases the number of false positives. |
|  |  |  | ≥4 | 85.7 | 50.3 | 5.9 | 99.0 | 48.5 |  |  |
|  |  |  | ≥5 | 71.4 | 59.1 | 6.0 | 98.3 | 40.5 |  |  |
|  |  |  | ≥6 | 71.4 | 70.5 | 8.1 | 98.6 | 29.5 |  |  |
|  |  |  | ≥7 | 71.4 | 79.8 | 11.4 | 98.7 | 20.5 |  |  |
|  |  |  | ≥8 | 71.4 | 85.0 | 14.7 | 98.8 | 15.5 |  |  |
|  |  |  | ≥9 | 71.4 | 91.2 | 22.7 | 98.9 | 9.5 |  |  |
|  |  |  | ≥10* | 71.4 | 93.8 | 29.4 | 98.9 | 7.0 |  |  |
|  |  |  | ≥11 | 57.1 | 95.3 | 30.8 | 98.4 | 6.0 |  |  |
|  |  |  | ≥12 | 42.9 | 95.9 | 27.3 | 97.9 | 6.0 |  |  |
|  |  |  | ≥13 | 42.9 | 97.9 | 42.9 | 97.9 | 4.0 |  |  |
|  |  | ‘Distress’ (minor/ major depression, adjustment disorder with anxiety (all criteria for GAD except duration of 6 months), panic disorder, specific phobia)  12/217 (5.5) | ≥3 | 100.0 | 37.6 | 8.6 | 100.0 | 59.0 |  |  |
|  |  |  | ≥4 | 83.3 | 49.8 | 8.8 | 98.1 | 48.4 |  |  |
|  |  |  | ≥5 | 75.0 | 58.5 | 9.6 | 97.6 | 40.6 |  |  |
|  |  |  | ≥6* | 75.0 | 69.8 | 12.7 | 97.9 | 30.0 |  |  |
|  |  |  | ≥7 | 66.7 | 80.5 | 16.7 | 97.6 | 20.3 |  |  |
|  |  |  | ≥8 | 66.7 | 85.9 | 21.6 | 97.8 | 15.2 |  |  |
|  |  |  | ≥9 | 66.7 | 91.7 | 32.0 | 98.9 | 9.7 |  |  |
|  |  |  | ≥10 | 66.7 | 94.1 | 40.0 | 98.0 | 7.4 |  |  |
|  |  |  | ≥11 | 41.7 | 94.6 | 31.3 | 96.5 | 8.3 |  |  |
|  |  |  | ≥12 | 33.3 | 95.6 | 30.8 | 96.1 | 7.8 |  |  |
|  |  |  | ≥13 | 33.3 | 97.6 | 44.4 | 96.2 | 6.0 |  |  |
| **Publication/ Country** | **Assessment** | **Mental health disorder; cases n(%)** | **Cut-point** | **Sens**  **(%)** | **Spec**  **(%)** | **PPV (%)** | **NPV (%)** | **Mis. (%)** | **Other results** | **Authors’ conclusions and recommendations** |
| Tran et al. (2012)  Vietnam | **Index test (version)**  EPDS, Zung’s self-rated anxiety scale (SAS), GHQ-12 (Vietnamese)  **Reference**  Structured Clinical Interview for DSM-IV (SCID), modules for depression, GAD and panic disorder  **Timing**  Pooled data: spanning antenatal (~28 weeks) and postnatal (~6 weeks) | Non-psychotic common mental health disorders (including major depression, dysthymia, GAD, panic disorder)  41/231 (17.7) | EPDS ≥3 | 78.1 | 56.8 | N/R | N/R | 39.4 | *AUC*  EPDS 0.767 (CI 0.679-0.855  Zung SAS 0.775 (CI 0.689-0.860  GHQ-12 0.792 (CI 0.712-0.871)  *Item endorse.*  N/R | The authors concluded that any of these measures could be used in men.  Cut-offs were lower in this study than in high-income settings, which authors propose may be due to cultural differences in emotional expression and the questions being framed as difference to their usual state, which may be insensitive to the experiences of those living in poverty and facing sustained adversity with enduring low mood.  The optimal cut-off for EPDS was higher in men than women, for Zung SAS was lower in men, and for GHQ-12 was the same. Reported that at respective optimal cut-offs, EPDS and Zung SAS gave comparable sensitivity and specificity for men and women, but that GHQ-12 had higher specificity in men.  Recommend use for screening “for PMCDs in men in research and at the primary health care level” in northern Vietnam (p.108). |
|  |  |  | ≥4 | 73.2 | 67.9 | N/R | N/R | 31.2 |  |  |
|  |  |  | ≥5* | 68.3 | 77.4 | N/R | N/R | 24.2 |  |  |
|  |  |  | ≥6 | 58.5 | 84.2 | N/R | N/R | 20.3 |  |  |
|  |  |  | Zung SAS ≥34 | 70.7 | 64.2 | N/R | N/R | 34.6 |  |  |
|  |  |  | ≥35 | 70.7 | 71.1 | N/R | N/R | 29.0 |  |  |
|  |  |  | ≥36* | 70.7 | 79.0 | N/R | N/R | 22.5 |  |  |
|  |  |  | ≥37 | 61.0 | 80.5 | N/R | N/R | 22.8 |  |  |
|  |  |  | GHQ-12 ≥1* | 75.6 | 74.7 | N/R | N/R | 25.1 |  |  |
|  |  |  | ≥2 | 51.2 | 92.1 | N/R | N/R | 15.1 |  |  |
|  |  |  | ≥3 | 36.6 | 96.3 | N/R | N/R | 14.3 |  |  |

Notes: * = optimal cut-off identified in paper; AUC = area under the curve; BDI = Beck Depression Inventory; CES-D = Centre for Epidemiological Studies-Depression Scale; CI = confidence intervals (all at 95%); EPDS = Edinburgh Postnatal Depression Scale; GHQ = General Health Questionnaire; HADS-A = anxiety subscale of the Hospital Anxiety and Depression Scale; item endorse. = item endorsement; mis. = misclassification, i.e. the percentage incorrectly classified; NPV = negative predictive value, refers to the proportion of fathers with negative test results (on the index test) who are correctly classified as non-cases; PHQ = Patient Health Questionnaire; PPV = positive predictive value, refers to the proportion of fathers with positive test results (on the EPDS) who are correctly classified as possible cases; ROC = receiver operating characteristics; sens = sensitivity, refers to the proportion of fathers who are cases (based on diagnostic interview) who are identified as possible cases (using the index test), i.e. the proportion of depressed fathers who are correctly classified; spec = specificity refers to the proportion of fathers who are non-cases (based on the index test) who are identified as being non-cases (using the index test), i.e. the proportion of non-depressed fathers who are correctly classified; ^W^ = weighted; Zung SAS = Zung's Self-rated Anxiety Scale
