# Supplementary material for: Assessing the Mental Health of Fathers, Other Co-parents, and Partners in the Perinatal Period: Mixed Methods Evidence Synthesis
Source: Front Psychiatry. 2021 Jan 12;11:585479. doi: 10.3389/fpsyt.2020.585479 (PMC7835428; doi:10.3389/fpsyt.2020.585479)
Supplement: Supplementary file 4 [file Table_4.docx]

**Supplementary Material Table 4: Characteristics and findings of acceptability studies (n=20)**

| **Publication / Country / Aims** | **Recruitment and selection** | **Sample details** | **Assessment (measures, details)** | **Relevant findings and recommendations reported by authors** | **Main limitations** |
| --- | --- | --- | --- | --- | --- |
| Bagge et al. (2017)  UK  *Aims*  Acceptability and feasibility of collecting outcome data with parents of very low birth weight (VLBW) infants in hospital neonatal intensive care unit (NICU), for research studies | *Recruitment*  Identified via admission records and approached on NICU, with input from nursing staff.  *Flow*  See feasibility comments  *Eligibility*  - Nursing staff deemed the baby to be stable. | *Sample size*  38 parents of VLBW infant (17 couples, 1 father, 3 mothers) and 36 parents of term infant (16 couples, 2 fathers, 2 mothers)  *Socio-demographics (fathers)*  VLBW fathers:  - Mean age 35.5 years (range 23-55)  - Time since birth: within early days/ weeks  - First-time fathers 38.9%  - Ethnicity: White British 94.4%  - Education: University 27.8%  Term fathers:  - Mean age 31 years (range 23-37)  - Time since birth: within early days/ weeks  - First-time fathers 83.3%  - Ethnicity: White British 94.4%  - Education: University 22.2% | CES-D, IES-R completed on the unit as part of a questionnaire pack, taking ~45 minutes (unclear whether self-completed or completed with the researcher) | *Feasibility*  - Approximately 60% of fathers that were approached consented to take part, of which approximately 80% completed and returned questionnaires (including some with partial completion).  - Accessing term parents was easier than VLBW parent (e.g. mothers of term infants were inpatients, fathers were on paternity leave).  - Flexible researcher hours were needed as fathers usually visited in evenings. Information sheets and consent forms were sometimes given to fathers by mothers.  *Acceptability*  Parents found it acceptable and easy to take part; scores were similar regardless of gender or group (VLBW/term). Some commented on time required (i.e. length) and on timing (e.g. emotions different at admission to discharge). Reasons for declining included physical barriers, imminent discharge, family disputes. One couple returned blank forms stating they were too intrusive. Both mothers and fathers identified need for emotional support for fathers.  *Recommendations*  - Research with this population needs flexible hours, shorter and simpler questionnaires (due to cognitive and affective load), designed for this group. | *Sampling*  - Unclear whether consecutive recruitment used  - Lack of ethnic diversity but this reflects local population  *Assessment methods*  - Unclear whether self-completed or completed with researcher  *Analysis*  - Acceptability was not reported by measure, limiting value for current review |
| Baldwin et al. (2019)  UK  *Aims*  Understand men’s experiences of first-time fatherhood, their mental health and wellbeing needs; including support from professionals | *Recruitment*  Invitation letter given to father or mother (if father absent) at new birth visit (~10-14 days postpartum), plus advertized in various settings (fathers’ groups, GP practice, health centres, children’s centres, nurseries, child health clinics)  *Flow*  25 fathers contacted researcher 🡪 21 met eligibility and all took part  *Eligibility*  - First-time resident fathers (biological or non-biological) with children <12 months.  - Excluded if non-English speaking, perinatal loss/baby loss, existing severe mental illness. | *Sample size*  21 fathers  *Socio-demographics (fathers)*  - Aged 20-60 years  - Time since birth: 14/21 ≤8 weeks; oldest 9 months  - First-time fathers 100%  - Relationship details: 2/21 not residing with partner at time of interview (although an inclusion criterion at assessment of eligibility)  - Ethnicity: 7 White British, 11 Asian, 2 Black, 1 White Other  - Education: University 19/21  - Employment: 100%; range of incomes | None | Theme of ‘health professionals and health services: experience, provision and support’.  - Some reported positive experiences with health professionals, where asked about their own wellbeing and made them feel included. Some felt excluded but accepted this, noting partner’s greater needs or professionals’ workloads.  - One noted professionals’ emphasis on child protection. GP as “professional of choice”, not maternity or health visiting.  Theme of ‘barriers to accessing support’.  - Fathers lacked knowledge of mental health support available to them.  - Most were not asked about their mental health and wellbeing in the perinatal period.  - Viewed services as focused on partner (mother).  - Other barriers included time/burden (for GP), stigma (family, friends, work), being culturally and socially unacceptable to discuss difficulties of fatherhood; questioned GPs’ training in dealing with fathers’ mental health.  Theme of ‘men’s perceived needs: what fathers want’.  - Although men felt excluded by professionals, they felt they too (like mothers) should be asked about their mental health and wellbeing and be offered the same level of support.  - ‘Most’ reported they would be willing to talk to professionals about their mental health; wanted information on signs and triggers; some would want to talk to professionals independently, away from partner; would only disclose if the appointment was clearly about them as well.  - Some thought access for mental health and wellbeing would be better if asked fathers questions, offered more flexible appointments (weekend/evening) and included father-focused information, preferably face-to-face or by telephone; this was relevant both in pregnancy and first few months after birth.  *Recommendations*  - Training for staff in paternal mental health.  - Routine mental health and wellbeing assessment and support for all fathers in the perinatal period, not only partners of women with mental health problems. | *Sampling*  - Reports maximum variation sampling but all eligible fathers took part  - Under-representation of young fathers, unemployed and lower socioeconomic status, although diverse for ethnicity  *Analysis*  - Some comments were unclear whether they explicitly referred to mental health or, for example, information for fathers more broadly |
| Clavenna et al. (2017)  Italy  *Aims*  Feasibility of routine screening with EPDS by family paediatrician at well-child visit (2-3 months postpartum) | *Recruitment*  Invited all parents attending clinic in 6-month study period at 1 site. Information about study was given at first visit (30-45 days postpartum).  *Flow*  See feasibility comments  *Eligibility*  - Attending second well-child visit (2-3 months postpartum). | *Sample size*  1,420 fathers (and 2,706 mothers)  *Socio-demographics (fathers)*  - Mean age 36.3 years (SD 4.8)  - Time since birth: 2-3 months  - First-time fathers 40.7%  - Relationship details: 93.1% married, 6.9% ‘single’  - Education: 30.1% primary school, 48.4% secondary school, 21.5% University  - Employment: 98.2% | EPDS (Italian version)  Study introduced by paediatrician at first clinic and asked to complete EPDS at second visit (2-3 months postpartum).  Self-completed in waiting room at clinic and returned to health professional. Parents were asked to complete separately and were “guaranteed privacy”.  *Onward management:* Parents scoring ≥13 were referred to a psychiatric service for management of postnatal depression. | *Feasibility*  - 38% fathers (and 73% mothers) participated in the screening.  - None of the 24 fathers (and only 11 of the 126 mothers) that scored above threshold agreed to attend a psychiatric service dedicated to the management of postnatal depression, although authors noted that parents may have accessed private psychiatry services.  *Recommendations*  - To be useful, routine screening of parents needs to be part of a wider initiative that includes access to specialist evaluation, involving multiple services (e.g. psychiatry, primary care family doctors and paediatricians). | *Data collection*  - Did not collect parents’ views, limiting value for current review  *Interpretation*  *-* The authors report that routine screening is feasible and can cover a “significant proportion” of parents; however, this is 38% fathers |
| Cole et al. (2018)  USA    *Aims*  Describe implementation of screening parents of newborns with prenatally diagnosed fetal anomalies in specialist hospital neonatal intensive care unit (NICU) by nurses. | *Recruitment*  In a 24-month period, obstetric nurses at a specialist obstetric unit within a paediatric hospital that had received training on screening asked parents to complete a screening tool within 24-72 hours of birth, before the woman’s discharge. Parents could opt out of completing and/or returning the tool.  *Flow*  802 women gave birth 🡪 753 were eligible and approached 🡪 725 mothers and 602 fathers  *Eligibility*  - Excluded if stillbirth or neonatal death occurred.  *Note*  Described as a highly specialized low-volume high-resource setting | *Sample size*  602 fathers (and 725 mothers)  *Socio-demographics (fathers)*  - Mean age 32.5 years (SD 6.1, range 15.9-59.9)  - Time since birth: within 24-72 hours  - Ethnicity: 64.4% White, 8.8% African American, 8.6% Hispanic, 4.7% Asian, 3.3% other.  - Education: 4.0% did not complete high school; 20.8% high school; 58.6% post-high school education (including vocational or University).  - Employment: 77.1% worked outside the home. | IES-R (all parents), CES-D (fathers), PDSS (mothers)  Measures were completed independently by parents, taking less than 10 mins, then returned to the obstetric nurse.  English or Spanish versions were available and hospital interpreters were used where needed.  *Onward management:* For those with elevated scores, mental health triage by clinical psychologist and referral to in-house mental health services was available.  All results, recommendations and interventions were documented in the maternal medical record. | - ‘Screening compliance rates’ were high (79.6% fathers and 96.5% mothers).  - Women’s partners are receptive to screening during a woman’s hospitalisation and screening parents of high-risk neonates is feasible.  - Partners in these circumstances may be at increased risk for perinatal mental health problems.  - Obstetric nurses with adequate resources and clinical training are well placed to implement mental health assessments and direct referrals for women and their partners in the early postpartum.  - Implementing within the context of in-house mental health services provided opportunity for discussion of psychological risk, provision of psychoeducation and resources.  - Staff engagement was facilitated by infrastructure and institutional support to address nursing concerns and patient safety issues as they arose.  - Increased workload of screening was off-set by streamlined follow-up planning (details not reported).  *Recommendations*  - There is a clinical need for assessing women and partners’ mental health.  - Screening at a later timepoint may help to define the impact of early identification and treatment. | *Sampling*  *-* Did not report how many of the 725 mothers had partners, therefore uptake in fathers unknown  *Data collection*  - Did not collect parents’ views, limiting value for current review |
| Currò et al. (2009)  Italy  *Aims*  Feasibility of routine screening with EPDS by family paediatrician at first well-child visit (15-20 days postpartum) | *Recruitment*  In an 11-month period, parents attending first well-child visit at a paediatric clinic were assessed using the EPDS during the visit. Those with high scores were invited to complete the EPDS at the second visit. Inclusion of fathers began in month 8 of the study.  *Flow*  See feasibility comments  *Eligibility*  - Excluded mothers with a history of depression; unclear about fathers. | *Sample size*  499 fathers (and 1,122 mothers)  *Socio-demographics (fathers)*  - Mean age 36.3 years (SD 5.5)  - Time since birth: 15-20 days  - Relationship details: 99.6% married or lived with partner  - Country of origin: 10.1% non-Italian (32.0% East Europe, 24.0% South America, 20.0% Asia, 12.0% West Europe and North America, 12.0% Africa).  - Education: 79.7% ‘school diploma or degree’  - Employment: 98.6% employed | EPDS (Italian, French, Spanish, English, Arabic, Punjabi, Singhalese versions; noting the last was not validated)  Completed “without any help”; setting not specified.  *Onward management:* After 5 weeks, high scoring parents (≥8 for fathers), completed a second EPDS. Any high scoring parents were examined by a psychiatrist who had to confirm the postnatal depression diagnosis. | *Feasibility*  - Mothers often attended the clinic alone.  - 499/501 (99.6%) fathers and 1,122/1,127 (99.6%) mothers completed the EPDS at the first visit.  - Of parents with high EPDS scores at the first visit, only 22/63 fathers and 147/298 mothers completed the EPDS at the second visit; this was described as high loss to follow-up.  - The EPDS took 2-7 minutes to complete; fathers mean 3.22 and mothers mean 3.28. It was quicker where parents were fluent in Italian (fathers mean 2.98, range 2-5; mothers mean 2.98, range 2-4) compared with ‘foreign’ parents (fathers mean 5.6, range 3-7; mothers mean 5.1, range 2-7). Amongst ‘foreign’ parents, it was quicker where the EPDS was translated into their mother tongue (fathers mean 4.33, range 3-5; mothers mean 3.73, range 2-6) compared with where it was not (fathers mean 6.00, range 5-7; mothers mean 6.00, range 5-7).  *Recommendations*  - “using a simple and standardized instrument, paediatricians are able to detect parents with higher risk of suffering from [postnatal depression]”.  *Notes*  Study examined agreement between EPDS score and diagnosis by psychiatrist but this did not involve structured diagnostic interview and therefore not included as a diagnostic test accuracy study. | *Data collection*  - Did not collect parents’ views, limiting value for current review  *Sampling*  - Reports that 5/1,127 mothers were excluded due to history of depression; this prevalence appears unusually low, raising questions about how this was assessed and defined |
| Darwin et al. (2017)  UK  *Aims*  Examine fathers’ views and experiences of their perinatal mental health and relevant resources; including mental health assessment | *Recruitment*  Via existing birth cohort where parents were recruited from maternity services/ antenatal appointments.  *Flow*  140 were invited 🡪 42 expressed interest in interview 🡪 22 invited to interview based on purposive sampling, aiming for maximum variation across mental health and wellbeing scores 🡪 19 took part.  *Eligibility*  - Previously completed mental health and wellbeing questionnaires in cohort study.  - Baby born at term (≥37 weeks gestation) and no serious health concern in mother/baby before discharge.  - Excluded same-sex parents due to focus on fathers. | *Sample size*  19 fathers  *Socio-demographics (fathers)*  - Mean age 33.1 years (SD 5.1, range 25-44)  - Time since birth: 5-10 months  - First-time fathers 14/19  - Reside with partner 100%  - Ethnicity: 18/19 White British  *Paternal mental health history*  - History not reported but range of mental health and wellbeing scores | Measures (PHQ-8, GAD-7, PHQ-15, List of Threatening Events) were self-completed during pregnancy and the postnatal period as part of the cohort study and used to sample participants for interview; views were not reported concerning these measures. | Theme on ‘legitimacy of paternal stress and entitlement to health professionals’ support’, sub-theme of ‘entitlement to health professionals’ support’.  Most men were receptive to and welcomed the suggestion of addressing paternal emotional wellbeing alongside maternal emotional wellbeing but perceived health services as under-resourced.  Most expressed concerns about “existing provision to support women’s mental health” and “compet[ing]” with meeting mothers’ needs. A minority of men did not feel the need to be asked about mental health, viewing this as unnecessary.  Fathers were conflicted regarding which health professionals could be involved in assessing partners’ mental health, noting fathers’ limited contact with services, and perceptions of midwives’ roles as focused on women and the pregnancy, and on physical rather than emotional health. Some questioned whether men would give ‘honest’ answers. Two participants accessed mental health support and did so via general practitioners, not maternity.  *Recommendations*  - There is a need for more research on male-specific measures and on the acceptability and effectiveness of assessing partners’ mental health together or apart. | *Sampling*  - Lack of diversity regarding ethnicity and socioeconomic status  *Analysis*  - Acceptability was not reported by measure, limiting value for current review |
| Fletcher et al. (2008)  Australia  *Aims*  Test a set of psychosocial questions with fathers, including ability to identify needs | *Recruitment*  Via antenatal classes at public/ private hospital.  *Flow*  1,043 surveys were given out 🡪 307 returned (response rate 29.4%) 🡪 acceptability interviews with 75 (24%) of those that returned survey (self-selecting by indicating agreement to be contacted on survey).  *Eligibility*  - Attending antenatal classes at study sites. | *Sample size*  75 fathers  *Socio-demographics (fathers - survey)*  - Mean age 33.19 years (SD 5.19)  - Timing: antenatal  - First-time fathers 92.2%  - Relationship status: 76.8% married  - Employment: 85.3% full time (85.3%); 91.2% professional, semi-professional,  or skilled occupations. | EPDS and 14 questions (e.g. relationships, finance); due to the assessment questions not being part of clinical care, questions about domestic violence, substance use and history of abuse were omitted to avoid “provok[ing] unnecessary distress” (p.28). | “None of the fathers interviewed by telephone were bothered by any of the questions” (p.30).  “Fifty-six (75%) thought that fathers would answer the questions honestly and 20 respondents (27%) volunteered that they had completed the survey with their wife or partner.” (p.30).  The fathers “had no difficulty with the questions posed and where the questions did raise issues they were commonly discussed with their wife or partner and described uniformly in positive terms (e.g., ‘‘made me think about things’’). This suggests that assessing psychosocial needs might be acceptable to fathers and their partners in the antenatal period.” (p.31).  *Recommendations*  - Further research is needed with a range of populations including culturally and linguistically diverse fathers. | *Sampling*  - Higher than average socioeconomic indicators (education and occupation)  - Characteristics were reported for survey respondents (n=307) but not the subsample that volunteered to be interviewed (n=75); acceptability amongst these 75 fathers may not be representative of the survey sample  *Analysis*  - Acceptability was not reported by measure, limiting value for current review |
| Fletcher et al. (2017)  Australia  *Aims*  Identify and describe instruments and procedures for screening fathers attending early parenting services (EPSs), and staff acceptability of screening fathers’ mental health | *Recruitment*  Approached managers at all 10 EPSs in Australia and asked to nominate 1 clinician and 1 supervisor.  *Flow*  1 manager declined the service being involved. All 18 staff nominated took part.  *Eligibility*  - Staff at EPSs in Australia.  *Note*  EPSs are specialist multidisciplinary services providing support to parents who are experiencing “complex parenting difficulties issues”; support may cover infant feeding, sleep and behavioural problems and maternal perinatal mental health concerns. | *Sample size*  18 professional staff from 9 EPS.  *Socio-demographics*  - Majority female (17/18)  - Range of job roles: 8 clinicians, 10 supervisors/ managers  - Years’ professional experience: mean 24.4 (SD 9.62, range 10-40)  - Time in current role: mean 7.3 years (SD 5.6, range 0.25-21 years)  - Range of formal qualifications: nursing 11 (31.4%),  child and family health nursing 11 (31.4%), psychology 3 (8.6%), social work 3 (8.6%), counselling 2 (5.7%), midwifery 2 (5.7%), other 3 (8.6%) | Various reported as being used within services (e.g. EPDS, DASS, K-10) | Broad categories of relevance were: identifying fathers’ mental health needs, the process for explaining the screening to fathers, screening for paternal depression when fathers are not present, addressing fathers’ mental health needs, and barriers to screening.  Most services (7/9) screened fathers for depression but there was a lack of consistency in approach used. Some were unclear regarding tools being used however the majority used EPDS; some used DASS; also reported were the K-10 and measures relating to parenting.  Whether screening occurred related to the service’s function and father’s involvement (e.g. primary caregivers, admitted to the service, actively participating). Some reported encouraging targeted screening where possible paternal depression was indicated in their history or family referral information, mentioned by the mother or if a nurse had concerns. Where EPSs were part of the universal system, screening occurred at the home visit <4 weeks postpartum.  Participants viewed screening as important and that routine approaches to screening would help to “normalize the process for both men and services.” (p.498). Services explained to fathers that screening was voluntary and respected fathers’ decisions to decline.  Services varied regarding the approach taken when father was not present at appointment, i.e. whether or not the mother was asked about the father’s mental health. Some women were asked in relation to family functioning, drug/alcohol use, safety and violence – they did not ask about mental health specifically. Professionals also noted the “tension…between mothers’ sharing of information, and confidentiality for fathers; additionally, some services did not accept the veracity of mothers’ descriptions of the father” (p.503).  Services varied as to whether they admitted fathers to be clients of the services (receiving support) or referred out of the service (with some depending on whether the clinician viewed the father’s mental health as related to the father-child relationship). Onward referrals (by formal letter or informal recommendation) included the general practitioner, employee assistance programmes, psychologists, counselling or social workers.  “Barriers to routine screening of fathers included work-family balance, service-level focus and characteristics of men themselves.” (p.504). Practical barriers included “service hours limiting time available to build a trusting relationship with the father, the prevalence of female staff and the lack of male-specific screening tools.” (p.504). Characteristics of men that could act as barriers included gendered perspectives on help-seeking and mental health, and men perceiving the service was for the mother’s mental health.  *Recommendation*  - EPSs provide a unique opportunity to address the mental health needs of fathers.  - A national approach is needed to develop father-specific screening guidelines.  - Research is needed to create specific tools for men that are appropriate and facilitates their participation. | *Sampling*  - Includes the perinatal period but also parents of older children (birth-preschool), limiting value for current review  - Authors noted absence of commentary on processes with minority groups, e.g. Aboriginal fathers or “those from culturally and linguistically diverse communities”  *Analysis*  - Acceptability was not reported in detail by measure, limiting value for current review |
| Freitas et al. (2016)  USA (international experts)  *Aims*  Reach expert consensus on the defining factors of paternal peripartum depression; including diagnostics, symptomatology, assessment.  Note: high consensus was defined as any coded response that received a mean score of ≥4 (using a 5-point Likert scale) and IQR of ≤1. | *Recruitment*  International experts identified via publicly available information; purposive sampling.  *Flow*  67 potential experts were identified 🡪 21 met inclusion and were invited 🡪 16 completed round 1 🡪 14 completed all rounds.  *Eligibility*  - Classed as expert if meeting 2 out of 5 criteria, including: working directly with fathers, peer-reviewed multimedia sources/ articles, facilitated training/ education, research project. | *Sample size*  16 partial completion 14 full completion  *Socio-demographics (14 completers)*  - Country: majority USA (9), one each from Canada, UK, Ireland, Australia, Poland  - Professional backgrounds: direct clinical or academic work, spanning psychiatry, psychology, sociology, nursing, social work, family therapy | Various mentioned as possible tools (e.g. EPDS, BDI, PDSS, GMDS, PHQ-9) | The categories ‘assessment protocol’ and ‘assessment tools’ obtained the lowest level of consensus across all questions (17.24%) compared to categories about e.g. symptoms and risk factors.  There was strong consensus that there is no set time for assessment because there is no guaranteed contact and assessment should therefore take place when the opportunity arises.  There was moderate consensus to assess during pregnancy and multiple times during the postnatal period.  “No specific screening tool reached moderate to strong consensus, although several came close” (p.133), listing the EPDS, BDI and PDSS. In the discussion, it reports that general depression tools (BDI, PHQ-9) and male-specific depression tools (GMDS) did not reach consensus for their use with the perinatal population. In addition, “the experts agreed that measures such as the [EPDS] may better detect a mother’s experience” (p.134). Authors report that the lack of consensus concerning assessment tools may reflect the consensus that there are several symptoms “unique to this population” (p.134).  There was strong consensus for a psychosocial interview to be conducted by someone who understands paternal perinatal mental health.  *Recommendations*  - A more sensitive assessment tool for fathers is needed.  - Public and professional awareness is needed to “integrate fathers as an equally important patient to consider in what is commonly seen as strictly maternal and child mental health” (p.135). | *Analysis*  - Although some tools were named in relation to consensus, acceptability was not reported by measure, limiting value for current review |
| Greening (2006)  UK  *Aims*  Assess the ‘And -how was it for you dad?’ questionnaire, designed to encourage men to think about and discuss how they feel, and to promote communication between health visitors and fathers. | *Recruitment*  Convenience sample, inviting the first 20 fathers that attended.  *Flow*  20 were approached 🡪 all 20 took part.  *Eligibility*  - Father on the author’s health visiting caseload. | *Sample size*  20 fathers  *Socio-demographics (fathers)*  - Aged 19-41 years  - Time since birth: 6 weeks  - First-time fathers: ‘mixture of first-time and experienced fathers’ (details not reported)  - Socioeconomic status: ‘mixed’ (details not reported) | Structured questionnaire (called ‘How are you – Dad?’) including experience of birth and fatherhood; no questions explicitly asked about mental health.  Completion unclear; intended to be given by health visitor at first contact following birth, then completed by fathers at 6 weeks to coincide with when mothers complete EPDS (locally called ‘How are you?’). Appears that fathers self-completed the structured questionnaire and the evaluation questions, then discussed both face-to-face with the health visitor; setting unclear. | 65% reported the questionnaire was helpful: 3 (15%) definitely helpful, 10 (50%) helpful to some extent, 1 (5%) not helpful, 4 (20%) not sure, 2 (10%) no comment.  60% reported the questionnaire had improved communication with their partner: 3 (15%) definitely improved, 9 (45%) yes to some extent, 5 (25%) not helpful, 1 (5%) not sure, 2 (10%) no comment.  20% reported the questionnaire had improved communication with their health visitor.  20% thought the questions should be more in depth. The participants “commented that [the questionnaire] should be more in depth, with questions more searching and similar to the [EPDS]” (p.187).  85% thought the questionnaire should be used in the future. Some example comments indicated that fathers are excluded, also noting that mothers are asked how they are, and that using such a questionnaire with men would help to make them feel included.  Overall, “nobody refused to take part and many welcomed the opportunity to express their feelings and emotions on becoming a parent” (p.186).  *Recommendations*  - A validated questionnaire needs to be developed “to assist men to think about how they feel on becoming a father” (p.187). | *Assessment methods*  - Unclear whether self-completed or completed with researcher  - Setting for completion unclear  *Reporting quality*  - Details on methods are limited  *Ethics*  - The author was responsible for first approach and for conducting the evaluation with fathers  *Reflexivity*  *-* Relationship between author and participants not adequately considered |
| Hammarlund et al. (2015)  Sweden  *Aims*  Explore child health nurses’ experiences of observing depression in fathers during the postnatal period and explore barriers. | *Recruitment*  Purposive sampling, recruited from 6 primary healthcare settings with units for child health care.  *Flow*  16 were approached 🡪 5 declined due to workload, 1 was sick 🡪 10 participated  *Eligibility*  - At least 1 year of experience of  working in child health care; across study sites, all nurses except for 1 fulfilled criteria. | *Sample size*  10 child health nurses  *Socio-demographics*  - All women  - Aged 40-59 years | None | Main theme: postnatal depression in fathers is “experienced as being vague and difficult to detect”.  Nurses felt unsure how to assess fathers’ health and wellbeing. This was linked to 5 related themes concerning challenges:  - limited experience of fathers with depression (including not seeing fathers regularly or having continuity);  - establishing contact with fathers; finding out about the father’s health status through the mother (and therefore perceiving this to be ambiguous and unreliable);  - lacking routines to assess fathers’ health (e.g. lacking a validated tool where the EPDS is used with mothers;  - routines being focused more on the mothers; needing ways to involve fathers);  - different “gendered-parenting practices” (i.e. gender attitudes influenced the daily work of the nurses e.g. assumptions that the mother would attend appointments; an emphasis of communication being with the mother rather than father; attitudes of staff and of parents).  *Recommendations*  - The key recommendation is that “Overall, more attention needs to be paid to postnatal depression in fathers where a part of the solution for this is that they are screened just like the mothers”.  - “Routines are needed to encourage the participation of fathers in [child health care]”.  - Screening tools for fathers need to be developed and validated.  - Screening tools need to be used as “an adjunct to clinical assessments when performing diagnoses”.  - Gender attitudes amongst health professionals need to be challenged. | *Sampling*  - Ability to take part depended on workload; acceptability amongst those unable to take part may differ |
| Massoudi et al. (2011)  Sweden  *Aims*  Investigate child health nurses’ perceptions of working with fathers; including identifying fathers with distress. | *Recruitment*  Random sampling of all nurses in Swedish child health care nationwide.  Distributed by post and used a reminder system.  *Flow*  499 were approached (random sample of 2,580) 🡪 348 (70%) participated  *Eligibility*  - Child health nurse in Sweden. | *Sample size*  348 child health nurses  *Socio-demographics*  - Professional training: primary health care, paediatric, midwifery  - Age: 60% aged ≥50 years, 10% ≤39  - Years’ experience: mean 13.3 (SD not reported, range 0.5-35 years)  - Work setting: public 90% and 10% private | None | 89% (302) estimated that it ‘only occasionally or practically never’ came to their attention that a father was distressed.  27% (91) stated they attempted by different means to identify fathers who were distressed. When asked to describe what they actually did, approximately one in five identified the first home visit as an opportunity to talk to fathers; many identified that they asked how he was feeling and adjusting; none used structured methods.  17.5% (60) stated they had offered supportive counselling to one or more fathers in the previous year, for a range of psychosocial difficulties.  Nurses with regular supervision on mental health issues and nurses with a paediatric specialisation were more likely to offer supportive counselling to fathers.  Reporting more broadly on fathers’ barriers to participation in child health care (not specific to mental health), nurses identified these to include financial/work-related barriers (including appointments being during the daytime) and nurses’ own responsibility in making fathers feel welcome.  *Recommendations*  - More awareness of gender bias is needed and need to develop ways to involve both parents in child health care and ask fathers about how they are feeling. | *Reporting*  - Some differences exist in questions asked in the 2004 and 2014 surveys (i.e. here and later Wells study), compromising the comparisons made, e.g. the earlier paper reports extra support for “any psychosocial difficulties” whereas the 2014 comparison describes this as “mental health problems”. |
| Oldfield & Carr (2017)  UK  *Aims*  Explore student health visitors’ and newly qualified health visitors’ perceptions of their role in supporting fathers when their partner had postnatal depression. | *Recruitment*  Describes as purposive sampling; no details reported.  *Flow*  Details not reported.  *Eligibility*  - Student on health visiting programme at 1 higher education institution or newly qualified health visitor.  - Have experience of managing/ observing management of postnatal depression with a father present at the family home. | *Sample size*  3 student or newly qualified health visitors  *Socio-demographics*  - Details not reported | None | Themes: lack of experience, gap in practice for support for fathers (when their partner has postnatal depression), searching for a solution.  Participants felt that paternal mental health was not part of their training both in theory and in practice, and they felt unable to adequately support fathers in practice.  Participants reported lacking confidence due to lack of exposure to fathers (i.e. practical experience and education during academic training). A lack of practical experience reflected fathers being at work, or that they may not stay in the room, even if present at the home.  Participants noted a potential ‘conflict of interest’ if wishing to support both parents equally; however, further details were not reported.  Participants viewed there to be a current lack of support for fathers and that there is no process for checking fathers’ mood.  Participants’ suggestions for support included online counselling, group settings, and one-to-one support as well as improving contact and engagement more broadly; suggestions concerning assessment were not reported.  *Recommendations*  - There is a need for change in education and practice, to ensure fathers’ need are met. | *Sampling*  - Unclear how participants were identified or approached  - Did not achieve intended sample size of four, reporting a “lack of response” from students and therefore extending to newly qualified professionals  *Analysis*  - Some comments were unclear whether they explicitly referred to fathers’ mental health or, for example, their support needs relating to their partner’s mental health  *Reporting quality*  - Details on methods are limited  *Reflexivity*  *-* Relationship between author and participants not adequately considered |
| Rominov et al. (2017)  Australia  *Aims*  Describe midwives’ perceptions and experiences of engaging fathers in perinatal services. | *Recruitment*  Via online newsletter to members of the Australian College of Midwives and email to a directory of midwives. Interest in interview was indicated in the survey.  *Flow*  Invited >5,000 🡪 106 participated in survey 🡪 13 participated in interview  *Eligibility*  - Registered midwife currently practicing in Australia with ≥6 months experience. | *Sample size*  Survey: 106 midwives  Interviews: 13 midwives  *Socio-demographics (survey)*  - Majority female (94.3%)  - Mean age 46.0 years (SD 10.4, range 22-64)  - Included public (72.6%) and private settings (27.4%)  - Mean 16.1 years working as a midwife (SD 10.5, range 0.5-37 years)  *Socio-demographics (interviews)*  - Majority female (12/13)  - Mean age 48.6 years (range 27-61).  - Work setting: public 9/13 (69.2%) and private 4/13, (30.8%)  - Mean 18.2 years working as a midwife (SD not reported, range 1-30 years)  *Note*  Interview participants were “generally representative” of the online survey sample | None | Thematic map provided showing factors relating to engaging fathers in maternity services: father, midwife and external.  Reported findings on views about fathers’ perinatal mental health:  - “…the majority of midwives reported feeling somewhat confident in asking fathers about their mental health and referring fathers to mental health services” (p.311).  - Perceived confidence in asking fathers about mental health:  10.64% extremely, 29.79% very, 40.43% somewhat, 11.70% slightly, 7.45% not at all.  - Perceived confidence in referring fathers to mental health services: 7.45% extremely, 30.85% very, 39.36% somewhat, 14.89% slightly, 7.45% not at all.  Most (83%) reported that they had not received any formal training about working with fathers; however, this was not specific to perinatal mental health.  All agreed it was important to receive extra training on fathers’ mental health.  The authors note that, “Midwives in the present study overestimated the extent to which fathers experience mental health difficulties, yet they did not feel confident asking about it.” (p.315).  *Recommendations*  - Research is needed with a range of health professionals across the perinatal period, to improve father-inclusive practice.  - “Fathers need to be embraced by all levels of the health care system as a critical component of perinatal care.” (p.315). | *Sampling*  - Online survey, unable to determine response rate  - Appears only 13 individuals in national workforce indicated interest in interview  *Analysis*  *-* The study is focused on engaging fathers and some comments are therefore not explicitly about mental health, limiting value for current review |
| Rowe et al. (2013)  Australia  *Aims*  Understand the anticipated needs and preferred sources of mental health information and support of men and women expecting their first baby; including the role of primary care in mental health care. | *Recruitment*  Via childbirth education classes in public and private hospitals  All eligible women and their partners were invited.  *Flow*  Details not reported.  *Eligibility*  - Nulliparous.  - English-speaking (sufficient fluency to self-complete survey and take part in group discussion).  - Could take part independent of partner involvement. | *Sample size*  16 fathers (and 22 mothers)  *Socio-demographics (fathers)*  - Mean age 31 years (range 20-37)  - Timing: antenatal  - First-time fathers 100%  - Relationship details: 7 (44%) married, 8 (50%) de facto; 1 (6%) divorced  - Country of birth: 7 (44%) born abroad  - Education: 7 (44%) University, 5 (31%) certificate or diploma, 2 (13%) secondary school, 2 (13%) part secondary school  - Employment: 100% employed; 80% professional, semi-professional,  or skilled occupations.  - Private health insurance: 9 (56%) | None | Men “understood…their own emotional wellbeing [was] not prioritised in current models of care and positioned themselves as marginalised and demeaned” (p.51). Alongside this, there were “diverse and gendered views about whether primary care providers should discuss mental health with parents of infants and willingness to complete written questionnaires or be referred for specialist mental health care” (p.46)  Theme on “The role of primary care in mental health care for new parents” with sub-themes of “routine enquiry” and “screening questionnaires”.  - In men, there was more resistance to both routine enquiry and screening questionnaires.  - Men perceived health professionals as not qualified to help with mental health, with some indicating they may feel less comfortable if experiencing distress; whereas women agreed they would like to be asked, that it was normalizing, reduced stigma, and gives “permission to express their needs” (p.50).  - Factors affecting men’s willingness to complete screening questionnaires included the questionnaire’s length, “how they were feeling at the time” (i.e. distress), perceived value of completion, and “competing priorities”. (p.51).  - “…the predominant discourse was that men’s role does not encompass display of emotional needs, nor is there permission for men’s needs to be recognised in services that are designed for mothers and babies” (p.51).  *Recommendations*  - There is a need for “increased provision of services that meet men’s needs and public understanding and acceptance of Australian integrated models of primary postnatal mental health care” (p.46). | *Sampling*  - Unclear how many parents were invited to take part or how the sample’s socio-demographic characteristics compare with the wider population |
| Samuel et al. (2005)  UK  *Aims*  Assess whether prospectively screening parents of children at a paediatric intensive care unit (PICU) for psychological vulnerability to PTSD would enable beneficial targeting of a subsequent follow-up clinic. | *Recruitment*  Parents of children consecutively admitted to PICU for ≥12 hours were invited to take part.  *Flow*  Invited 278 families (out of 645 admissions) 🡪 145 families (52%) were consented.  *Eligibility*  - Excluded if child died, palliative care, admission for nonaccidental injury, readmission in study period, or deemed inappropriate (e.g. ‘social problems’).  - Some were missed because researcher was not available. | *Sample size*  209 parents (of 145 children)  *Socio-demographics (fathers)*  - Details of parents not reported.  - Mean age of child at discharge 0.89 years (median 0.16, IQR 4.50) | Post-traumatic Adjustment Scale (PAS; with amended wording e.g. I thought my child was about to die) completed during stay; distributed and collected by assistant psychologist; measure took 5 minutes to complete. Acceptability was assessed at 6 months.  *Onward management:*  High-scoring parents were randomized to the intervention (follow-up clinic, 2 months after discharge) or to treatment as usual. | Authors described the PAS as acceptable to parents: 85% of 124 completing acceptability did not report any distress in completing the measure.  Only 14/38 (37%) high-risk parents attended the follow-up clinic appointment they were offered; gender not reported. Those that did and did not attend did not differ on gender, PAS score, or admission length; those attending had older children. Of 28 invited to clinic who responded, 96% appreciated the offer. Barriers to attending clinic (reported by 19 non-attending parents) included work commitments and childcare.  *Recommendations*  - The PAS may be useful in research and clinically to determine which parents are most likely to need support after their child’s PICU admission.  - Screening may enable more efficient targeting of support but research is needed on how best to provide follow-up intervention for this population.  - As part of post-traumatic stress, parents may avoid reminders to their traumatic experience and this may therefore be a barrier to engaging with support. | *Sampling*  - Includes the perinatal period but also parents of older children (birth-preschool), limiting value for current review  - Only 124/209 that completed the measures went on to complete the acceptability questions  *Analysis*  - Results are not reported by gender |
| Schuppan et al. (2019)  Australia  *Aims*  Explore with at-risk men acceptability of screening for paternal mental health concerns and their help-seeking behaviours. | *Recruitment*  Via Women’s and Children’s Hospital, either from Parent Education classes or antenatal clinic waiting room, providing a flyer with webpage link to complete the survey. Any that were eligible were invited to interview.  *Flow*  Number approached not reported 🡪 16 completed online questionnaire 🡪 11 met eligibility and invited to interview 🡪 8 took part  *Eligibility*  - Male.  - Aged > 18 years.  - Able to converse in fluent English.  - >28 weeks gestation.  - >5 on EPDS and/or  current/past depression or anxiety diagnosis from a health professional. | *Sample size*  9 fathers  *Socio-demographics (fathers)*  - Mean age 38.13 years (SD 13.9, range 24-64)  - Timing: majority late pregnancy, some early postpartum  - First-time fathers 5/8 (62.5%)  - Relationship details: all living with partner  - Country of birth: 6 Australia, 2 New Zealand, 1 UK  - Ethnicity: 5 Australian, 1 New Zealander, 2 European  - Education: 5 University, 2 trade / professional qualification; 1 secondary education  - Socioeconomic status: 1 ‘just getting along’, 5 ‘reasonably comfortable’, 2 ‘very comfortable’  *Paternal mental health history*  - Current depression diagnosis 0 (0%)  - Prior depression diagnosis 3 (37.5%)  - Current anxiety diagnosis 0 (0%)  - Prior anxiety diagnosis 2 (25.0%) | EPDS, completed online. Survey including EPDS and demographics took 6.5 minutes to complete, on average. | Overall, “…routine screening is desired, but acceptability is influenced by perceptions of its intention and possible outcomes. Findings also suggest that barriers to men’s perinatal help-seeking are likely to be minimized by increased awareness and normalization.” (p.1)  Theme: acceptability of screening with EPDS  - *Experience of the scale -* largely positive; spoke about *ease, relevance*, and *feel* of the measure (e.g. number of questions, multiple-choice, anonymity). Suggested giving the option for paper or online completion.  - *Screening as awareness raising –* help-seeking can be seen as an issue of “*personal responsibility”* which could resist routine screening but was broadly compatible, seeing increased awareness of own symptomatology as a “help-seeking prompt”  - *Perceived intention and interpretation –* lack of comprehensive explanation or interest on behalf of a health professional would increase stigma, suspicion and dishonesty; need to acknowledge external stressors; be aware men may be fearful of others’ reactions  *- Normalizing –* screening helps to reduce stigma and normalize emotional experiences  Theme: help-seeking for mental health concerns in the perinatal period  - *Stigma* – reluctance to be seen as weak or feel vulnerable  - *Role -* primarily to support and therefore would not want to be seen as weak or not fulfilling role  - *Spare part -* included that fathers had not been asked about their experiences, which authors later describe as not having “permission” for their needs to be recognized in services focused on mothers and babies.  *Recommendations*  - Future research should “not only ask “should we screen” and “is screening acceptable” but further “*how* should we screen” and “*what makes* screening acceptable”” (pp.16-17).  - “Future research and clinical practice should account for complexities raised by men’s perceptions of health care services.” | *Sampling*  - Number approached and response rate not reported  - Characteristics reported for 8 recruited to study but pilot interview was also used in analysis  *Analysis*  - Accounts indicated that fathers welcomed anonymity however this would not apply if assessment was part of clinical practice |
| Ståhl et al. (2020)  Sweden  *Aims*  Explore child health nurses’ experiences of performing parental interviews with nonbirthing parents | *Recruitment*  Child health nurses in one region, receiving 1-day training on how to conduct parental interviews with nonbirthing parents at ~3-5 months postpartum; data collection was conducted at follow-up several months later, when interviews had been implemented.  *Flow*  All 116 nurses attending training in the study period were invited to take part 🡪 11 participated.  *Eligibility*  - Child health nurse attending training. | *Sample size*  11 child health nurses  *Socio-demographics*  - Details not reported | Whooley questions, followed by EPDS in the event of a positive response; completed within a comprehensive ‘parental interview’. | Overall theme: “a constant challenge in promoting child welfare”, with two main categories: “Establishing a relationship with the nonbirthing parent” and “A deepened family perspective”.  Although a positive experience, parental interviews with nonbirthing parents offered “both possibilities and obstacles”. Reported challenges include: having close contact with both parents and feeling caught between them; feeling worried about handling difficult information. Some experienced challenges with keeping parents’ viewpoints apart, honoring confidentiality when feeling the other parent should know the information, or that information given by one parent could affect the interview with the other. Some felt they make lack education or competence to manage the topics encountered and feeling like a mediator between the parents; noting these dynamics would be avoided in other therapeutic relationships.  Prior to training, some had offered individual interviews to nonbirthing parents when they noticed “problems in the family” or parents had requested it. Having the interview’s “planned conversational guide” was described positively and contrasted with previously experiences of having “loosely organised conversations”, even if the EPDS was used previously.  “The nurses described how they had only positive responses from the nonbirthing parents they had interviewed, in this case, fathers, and those that were offered and accepted the individual interview were satisﬁed, but at the same time somewhat surprised to be included and noticed.” (p.142)  Nurses valued the message given to society and parents by having individual interviews, indicating the importance of nonbirthing parents and helping them to feel included.  “The invitation for the parental interview was mostly given during a normal child visit or during home visits, but sometimes, the mother had suggested that the father could use a special visit. On some occasions, fathers themselves ﬁrst accepted the invitation but later cancelled the interview, either directly to the CHS nurse or through the mother some time before the interview, arguing that they did not feel a pressing need for it. This is a phenomenon the CHS nurses recognized from when implementing the maternal PPD screening. The CHS nurses expressed an expectation that participating in the interviews would, in time, be seen as more common and natural, as was shown to be the case for the mothers.” (p.142)  *Recommendations*  - Broadening the duties and responsibilities of nurses to include non-birthing parents in mental health screening requires proper guidance and education, resources, onward referrals, support from psychologists, and sufficient time, for the discussions to be possible and meaningful.  - Training needs to address potentially difficult situations, .g. feeling caught between parents/risk acting as a mediator, managing confidentiality, knowledge/suspicion of violence/abuse (of the child or between partners).  . | *Sampling*  - Taking part was restricted to those who had not yet conducted interviews; reasons for not yet having conducted interviews included: lack of time/resources, waiting for colleagues to have the training; those who took part may therefore have experienced fewer barriers  - Sample size was smaller than planned, due to challenges with recruitment  *Analysis*  - Acceptability was not reported by measure, limiting value for current review |
| Wells et al. (2017)  Sweden  *Aims*  Investigate child health nurses’ perceptions of working with fathers, including identifying fathers with distress; making comparisons between 2004 (Massoudi study) and 2014. | *Recruitment*  Questionnaire distributed to nurses at local conferences in one area.  *Flow*  390/484 nurses in the area attended conferences (main reason for not attending: working part-time, sick leave, parental leave) 🡪 363/390 participated.  *Eligibility*  - Child health nurses in local area attending conferences. | *Sample size*  363 child health nurses  *Socio-demographics*  - Professional training: primary health care, paediatric, midwifery  - Age: 50% aged ≥50, 21% ≤39  - Years’ experience: mean 11.4 (SD not reported, range <1 year -40 years)  - Work setting: public 90% and 10% private  *Note*  Although regional in 2014 and national in 2004, sensitivity analysis of regional data from 2004 indicated similar trends. | None | Compared to 2004, fathers were viewed more equally by nurses but many nurses did not feel they had the skills to support fathers; more support was provided to both parents (compared to 2004) but mothers still received most of the parenting support.    It was more common to address emotional distress and mental health issues with fathers in 2014 compared with 2004. However, in 2014, “many… [still] seemed to be unaware of paternal distress and other mental health issues in fathers” (p.1306).  In 2014, 75% of nurses estimated it only occasionally or hardly ever came to their attention whether a father had mental health difficulties, compared with 89% in 2004. In 2014, when nurses noticed fathers’ mental health issues, 63% reported doing something extra to acknowledge these fathers, compared with 27% in 2004. In 2014, 38% had provided extra support (e.g. supportive counselling) to ≥1 of fathers for their mental health problems in the last year, compared with 17% in 2004. All were statistically significant differences.  In 2014, nurses provided supportive counselling to fathers as often for his own depression or other mental health problems (19%, n=68) as for his partner’s (21%, n=75).  *Recommendations*  - Organisational changes are needed within child health to provide more inclusive support to fathers; e.g. addressing organisational barriers (e.g. working hours, caseload) and personal barriers (e.g. nurses’ attitudes, nurses’ competence in supporting fathers, especially with mental health).  - Notes that mothers have a postnatal visit that includes screening for postnatal depression and “identifying distressed fathers during a father-only visit” might benefit father and child outcomes (p.1307). | *Reporting*  - Some differences exist in questions asked in the 2004 and 2014 surveys (i.e. here and later Wells study), compromising the comparisons made, e.g. the earlier paper reports extra support for “any psychosocial difficulties” whereas the 2014 comparison describes this as “mental health problems”. |
| Whitelock (2016)  UK  *Aims*  Examine why health visitors do not screen both parents for postnatal depression | *Recruitment*  Purposive sample at one Trust (study site).  *Flow*  Details not reported.  *Eligibility*  - Health visitor at the study site.  - Regularly using the EPDS to assess mental health (with mothers). | *Sample size*  12 health visitors  *Socio-demographics*  - Details not reported | EPDS | Themes: “Constraints health visitors feel prevent them from engaging with fathers”; “Why men do not engage well with the health visiting service”  Health visitors reported anxieties about offering screening and emotional support to fathers which they felt prevented them (p.316):  - no training related to working specifically with fathers;  - no training related working with men who have poor mental health; - fears for own safety when with men;  - lack of confidence in working with fathers;  - busy caseloads and time factors;  - health visiting culture, perceiving as a ‘mother and child’ service;  - no workplace policies about screening men.  Professionals viewed that fathers do not engage due to (p.315):  - individual culture and beliefs;  - own or family expectations regarding their role as a new father;  - gender, with health visitors perceived as a ‘mother and child’ service, leading to feelings of exclusion from health services;  - stigma of talking about poor paternal mental health.  Linked to this, some had concerns that asking fathers about their mental health may cause offence due to individual culture, religion or personal beliefs.  “One participant said that she would feel comfortable completing the EPDS with fathers, although suggested that some of the words used would need to be changed to make them more ‘man-friendly’.” (p.316).  Although some viewed screening fathers using the EPDS as potentially beneficial, most rejected the proposal due to concerns about personal safety (e.g. some felt vulnerable if working alone with men, especially if there were unknown mental health concerns) and viewed it as impractical because caseloads would become unmanageable. Many thought that health visiting should continue to be child- and mother-focused because fewer fathers engage.  *Recommendations*  - Training on paternal mental health for health professionals including pre-registration and in practice.  - Mandatory training on conflict resolution and lone worker training should include focus on mental illness and men.  - Both parents’ mental health should be ‘screened’.  - Health visitors should prompt mothers with depression to encourage their partners to be screened.  - There should be online access to health visiting to support fathers.  - There is a need to “evaluate and reshape maternal mood assessment policies to include fathers where histories of poor mental health or predisposing factors for depression are known” (p.318).  - “Even if fathers are not present during new birth or antenatal visits, mothers should be asked whether the baby’s father has any known mental health concerns and offered an assessment for postnatal depression screening.” (p.317).  - Screening of fathers should be between 3 and 6 months following birth and “not at the same time as the maternal mood assessment, to provide individual assessment, privacy and support and to ensure that both parents do not influence each other’s results.” (p.318).  - There is a need to address health visiting culture and tradition to meet families’ needs. | *Sampling*  - Unclear how many staff were invited to take part  *Reporting quality*  - Details on methods are limited  *Reflexivity*  *-* Position of author and relationship between author and participants not adequately considered (e.g. the author strongly advocates for routine assessment of all fathers using the EPDS and in the background, states that on the basis of previous research “the EPDS would be perceived an acceptable, routine assessment tool used for all new fathers”, p.315) |

Notes: BDI = Beck Depression Inventory; DASS = Depression Anxiety Stress Scales; EPDS = Edinburgh Postnatal Depression Scale; GAD = Generalized Anxiety Disorder; GMDS = Gotland Male Depression Scale; IQR = inter-quartile range; K-10 = Kessler 10-item scale; PHQ = Patient Health Questionnaire; PDSS = Postpartum Depression Screen Scale; SD = standard deviation
